# Dynamically assembled magnetic nanoparticles in a phase transitional matrix for reconfigurable electronics

**DOI:** 10.1126/sciadv.adw6611

**Published:** 2025-09-12

**Authors:** Min-Gyu Lee, Seong-Yu Choi, HyunJae Yoo, Jae-Man Park, Younghoon Lee, Yun Hyeok Lee, Yong Eun Cho, Sungsoo Lim, Hakjun Lee, Jeong-Yun Sun

**Affiliations:** ^1^ Department of Material Science and Engineering, Seoul National University, Seoul, South Korea.; ^2^ Department of Chemical and Environmental Engineering, Yale University, New Haven, CT 06520, United States.; ^3^ Department of Mechanical Engineering, College of Engineering, Kyung Hee University, Yongin, Republic of Korea.; ^4^ Research Institute of Advanced Materials (RIAM), Seoul National University, Seoul, South Korea.

## Abstract

The structure of an electronic device is predetermined at its birth, necessitating new designs and fabrication processes for alternative functions. The advent of reconfigurable electronics, modifying its circuits after manufacture, has unlocked the potential for devices to perform adaptive roles as needed. However, the trade-off between the degree of freedom to reform its structure and electrical stability restricts its potential roles, diminishing the system’s significance. Here, we present a reconfigurable assembly of magnetic nanoparticles in a phase transitional matrix (RAMP) system capable of seamlessly transforming their structure with robust electrical junctions. Nanoparticles form conductive percolation under a precisely patterned magnetic field. Within a phase transitional matrix, junctions between nanoparticles are tightened, enhancing electrical performance during transitions. We demonstrated in situ electrical switching and high-resolution alternating current electroluminescence display using the RAMP system. With enhanced reconfigurability and electrical reliability, we anticipate that the RAMP system will suggest a previously unexplored approach to on-demand electronics.

## Teaser

A phase transitional matrix facilitates the reconfiguration of the conductive percolation and enhances its electrical performance.

## INTRODUCTION

Reconfigurable electronics can modify its circuit after fabrication to change the function on demand. This adaptability enables a versatile, efficient, and sustainable approach to circuit design, rendering reconfigurable electronics vital to emerging advanced technologies (*1*). A fundamental requirement for reconfigurable circuits is the ability to reform the configuration of conductor units, enabling precise and dynamic adjustments to circuit properties (*2*–*9*). Conductive nanomaterials such as nanoparticles, nanowires, and two-dimensional materials have emerged as ideal candidates to facilitate the design of reconfigurable circuits (*10*–*18*). Their ability to be easily assembled into percolation networks makes them highly suitable for forming adaptable electrical pathways.

Since the electrical performance of a percolation network is predominantly influenced by the junction resistance (fig. S1), it is imperative to stabilize the contact resistance at these junctions effectively (*19*–*21*). To attain robust junction stability in percolation networks, strategies such as self-healing (*2*, *15*, *22*–*24*), hydration/dehydration (*25*, *26*), surface modification (*13*), and junction welding (*27*, *28*) have been attempted. However, improving junction stability tends to compromise the behaviors of nanomaterials, thereby limiting the degree of reconfigurability. Thus, an inherent trade-off exists between high reconfigurability and robust junction stability. We believe that this trade-off can be resolved using a phase transitional material, which is generally used in flexible and wearable electronics due to its innate tunability, conformability, and biocompatibility (*29*–*31*)_._

Here, we developed reconfigurable assembly of magnetic nanoparticles (MNPs) in a phase transitional matrix (RAMP), achieving both seamless control and stable retention. The phase transitional matrix enables switching between two distinct states: a flowable sol state to reconfigure an electrical circuit and a solid gel state to stabilize the circuit. Furthermore, because the conductive percolations could be strengthened by depletant exclusion and volumetric contraction during the phase transition, the quality of the percolation is enhanced. The related mechanisms of the RAMP system are discussed, along with a demonstration of a reconfigurable ac electroluminescence (ACEL) display as a potential application.

## RESULTS

### Reconfigurable assembly of magnetic nanoparticles in a phase transitional matrix

To implement the RAMP system, we used ferromagnetic iron nanoparticles as reconfigurable building blocks and conductive units simultaneously for our system (*32*) (fig. S2). A phase transitional matrix was prepared using 12-hydroxystearic acid (12-HSA) as a low–molecular weight gelator (LMOG) and octadecene (ODE) as a solvent. The 12-HSA enables thermally reversible sol-gel transition near 65°C, while ODE facilitates phase mobility during particle rearrangement (figs. S3 and S4). The matrix exhibits moderate mechanical properties at room temperature (fig. S5) and remains electrically nonconductive.

**Fig. 1.**
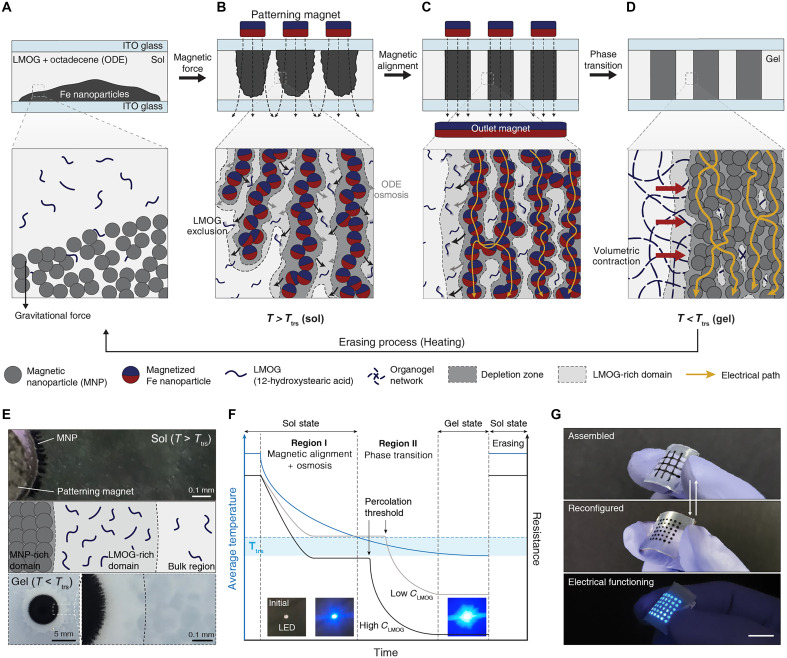
Concepts for reconfigurable assembly of magnetic particles via magnetic field alignment and phase transition. (**A** to **D**) Schematic illustration of reconfigurable MNP assembly process. (A) Above the *T*_trs_, the MNPs are settled down in the LMOG + ODE solution molded by the ITO electrodes and the spacers. (B) The emanating magnetic field from the patterning magnet attracts the MNPs and creates overlapping depletion zones of MNPs, resulting in LMOG exclusion and ODE osmosis. (C) The outlet magnet aligns the magnetic field linearly and uniformly, resulting a fine arrangement of particles as desired. An electrical pathway forms through the particle assembly. (D) During the phase transition of LMOG + ODE solution, the percolation of MNPs is reinforced by a volumetric contraction. The percolation is maintained without the external magnets after the transition. The assembly can be erased by elevating temperature above *T*_trs_. (**E**) LMOG exclusion creates an LMOG-rich domain near the MNP assembly. (**F**) Schematic illustration of the resistance evolution during magnetic alignment and sol-gel phase transition. Region I: As the percolation of nanoparticles formed by magnetic alignment and osmosis, the electrical pathway has constructed. When volumetric concentration of MNP is fixed, the level of the plateau resistance is determined by the concentration of LMOG. Region II: During the phase transition, the volumetric contraction induced by the transition reinforces the percolation of nanoparticles, resulting a lower electrical resistance. The inset images show a change of brightness of a light-emitting diode (LED) connected with the percolation. (**G**) A reconfigurable electrode based on RAMP functionalizes an electroluminescent device using ZnS. Scale bar, 1 cm.

The RAMP process begins with these materials encapsulated between two indium tin oxide (ITO) electrodes and confined laterally by acrylic spacers, forming a sealed chamber above the transition temperature (*T*_trs_) (Fig. 1A). Without any external magnetic field, iron nanoparticles are settled down within the sol-state matrix due to the gravitational force. To apply an external magnetic field as desired, we fabricate patterning magnets through a polymer casting method with a silicone-based elastomer and neodymium iron boron (NdFeB) particles (fig. S6). When the fabricated patterning magnet approaches to the top substrate, the settled nanoparticles are attracted toward it by magnetic force. The formation of the particle assembly under the magnetic field occurs simultaneously with the overlapping of the depletion zones surrounding each nanoparticle (Fig. 1B, fig. S7, and text S3 for details) (*33*). This overlapping expels 12-HSA from the particle percolation, resulting in an LMOG-rich domain around the particle assembly. The uneven LMOG concentration causes the solvent to diffuse out from the assembly by osmosis (*34*–*36*).

However, because of the spreading magnetic field from the patterning magnet and the edge effect where its edges exhibit a higher magnetic flux density than the center (fig. S8), particles cannot be controlled as desired to form electrically conductive pathway. To achieve precise control by overcoming these characteristics of the magnetic field, we introduce an outlet magnet to the opposite side of the patterning magnet. The outlet magnet aligns the previously dispersing magnetic field lines straight and makes the magnetic flux density uniform (fig. S9). Consequently, nanoparticles are assembled into the desired pattern by the aligned magnetic field (Fig. 1C and movie S1). Although this magnetic field alignment technique is a conventional method for controlling magnetoresponsive materials, such as ferrofluids (*37*) and magnetorheological elastomers (*38*–*39*), previous works have primarily focused on controlling the bulk properties of the materials. In contrast, our approach enables the selective control of electrode patterning by precisely placing nanoparticles at desired locations. Moreover, our system introduces a reversibly reconfigurable platform enabled by a phase transitional matrix. This capability is particularly substantial for applications in reconfigurable engineering and electronics. An electrical pathway forms through the percolated nanoparticles by magnetic alignment and osmosis in the sol state. As the temperature decreases below the *T*_trs_, the gelation initiates at heterogeneous sites. The stable formation of a gel network through intermolecular hydrogen bonds between LMOGs causes the matrix to slightly shrink (Fig. 1D). The resulting percolated structure was visualized using x-ray microscopy (see movie S2), confirming the integrity of the particle assembly. This contraction enhances the robustness of the conductive network by tightening interparticle junctions, thereby improving the overall electrical performance. Within the gel, the nanoparticle percolation functions as a patterned electrical pathway, remaining stable even after the magnet is removed or in the presence of external magnetic interference up to 2.5 T (fig. S10). The whole process can be repeated multiple times by increasing the temperature to transition the matrix from a gel state to a sol state.

In the sol state, although a concentration gradient of LMOGs was formed by depletion, both the domain adjacent to the nanoparticle assembly and the bulk region remain transparent above *T*_trs_ (Fig. 1E). However, after gelation, a distinct difference in opacity was observed between the region near the MNP-rich domain and the bulk region. Observing that organogels with higher LMOG concentrations appear more opaque (fig. S11), we could infer that LMOG molecules are excluded from the MNP-rich domains due to depletion effects. This interpretation is further supported by differential scanning calorimetry (DSC) analysis, which confirms the localized accumulation of LMOG near the nanoparticle assemblies (fig. S12).

The resistance through nanoparticle percolation changes as the RAMP process progresses (Fig. 1F). A composite of nanoparticles and LMOG solution, heated to around 90°C, is slowly cooled at room temperature (fig. S13), exhibiting two distinct stages of resistance change: (i) Upon the application of the aligned magnetic field, the resistance through the simultaneously formed nanoparticle assembly rapidly drops and stabilizes. When the particle content is held constant, the level of the resistance plateau is determined by osmosis induced by the localized concentration difference of LMOG. As the LMOG contents increase, a greater concentration gradient is created, which, in turn, strengthens the depletion force, leading to lower resistance (*33*). (ii) As gelation progresses after *T*_trs_, the volume of the matrix begins to contract slightly, squeezing the particle percolation at the junctions between particles. After the critical degree of gelation, the locally increased density of the particle assembly surpasses the percolation threshold, referring to the stage during the phase transition at which the nanoparticle network attains long-range connectivity and exhibits a sharp enhancement in electrical performance (*40*). Furthermore, it is observed that the concentration of LMOGs influences the kinetics of gelation, resulting in different times to overcome the critical percolation threshold.

We fabricated a patterned electrode that is reconfigurably modifiable with the RAMP system (Fig. 1G). The fabricated samples are soft and self-standing, functioning as electrodes to operate a reconfigurable ACEL display.

### Formation of nanoparticle percolation via magnetic alignment

**Fig. 2.**
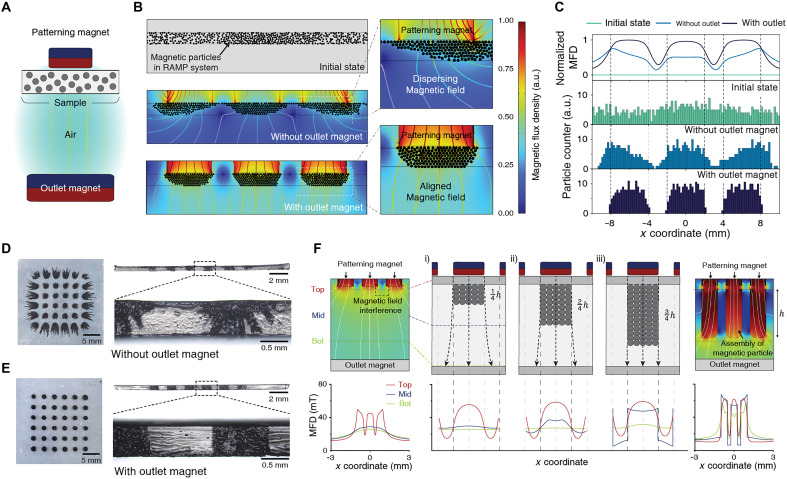
Patterning of MNP under an aligned magnetic field. (**A**) Schematic illustration of an FEM simulation to demonstrate a distribution of magnetic particles in the RAMP system. (**B**) FEM simulation depicting the behavior of the magnetic particles at the initial state, without an outlet magnet, and with an outlet magnet. a.u., arbitrary units. (**C**) Normalized magnetic flux density (MFD) and particle counter histograms. The magnetic flux density is normalized by the maximum value within the system. (**D** and **E**) Optical microscope images displaying the configurational difference of magnetic particles without (D) and with (E) an outlet magnet. (**F**) Propagation of the assembly of magnetic particles forms a magnetically permeable pathway, enhancing resolution in the RAMP system. For [(B) and (C)], the magnetic flux density is normalized by the maximum value within the system.

Finite element method (FEM) simulations are conducted to quantitatively understand the effect of magnetic alignment in the RAMP system (see text S1 for details of the computational simulation model). Schematic illustration for the FEM simulation is shown in Fig. 2A. Five hundred magnetic particles are randomly spread at the initial state (Fig. 2B). Without the outlet magnet, magnetic pathways from the patterning magnet disperse in every direction. This inhomogeneous magnetic field results in 23.2% of the magnetic particles not stacking as desired. However, after introducing the outlet magnet at the bottom of the sample, the magnetic fields are aligned with uniform density, reducing the edge effect significantly. As a result, 97.6% of the particles are stacked below the patterning magnets. The magnetic flux density graph and particle counter histograms of the initial state, without an outlet magnet, and with outlet magnets are demonstrated in Fig. 2C. Detailed geometrical settings for the simulation and magnetic flux density graphs are depicted in fig. S14. Similar to simulation results, the images of optical microscope also verify the effect of the outlet magnet on magnetic alignment (Fig. 2, D and E). Samples with outlet magnets demonstrate uniformly packed magnetic particles, showing clear differences compared to those without outlet magnets. Following these settings, character patterning with optimal resolution is obtained (fig. S15). In addition, simulations with a pair of patterning and outlet magnet are conducted to optimize the alignment of the magnetic field and visualize the behavior of MNPs under magnetic fields (fig. S16 and movie S3). On the basis of the simulation results, the optimized distance and magnetic strength of the magnets are determined to achieve high-resolution patterning (fig. S17). As a result, the patterning resolution achievable through magnetic alignment is ~500 μm (fig. S18).

However, the alignment of magnetic fields is not achieved by the magnets alone. Without the magnetic particles, the magnetic fields interfere with each other as the size and distance of the patterning and outlet magnets decrease (Fig. 2F, left, and fig. S19). Here, we hypothesize that to achieve high-resolution patterning of 1 mm or less, not only are the patterning and outlet magnets needed but the assembly of magnetic particles as a magnetic pathway is also crucial. To verify this hypothesis, we conduct FEM simulations with a series of assemblies having a relative permeability of 100 (Fig. 2F, middle, and fig. S20). The schematic illustration of Fig. 2F depicts the influence of the assembly on the magnetic fields. The difference in average magnetic flux density under the patterning magnets between the top and middle points, as well as between the top and bottom points, is inversely proportional to the height of the assembly. By introducing the assembly that fully fills the RAMP system, the average magnetic flux density difference between the top and middle points decreases from 38.66 to 6.55%, while the difference between the top and bottom point decreases from 45.38 to 19.01% (Fig. 2F, right). Collectively, magnetic particles create a magnetically permeable pathway itself through their interaction with the aligned magnetic field.

### Analysis and characterization of RAMP during phase transition

**Fig. 3.**
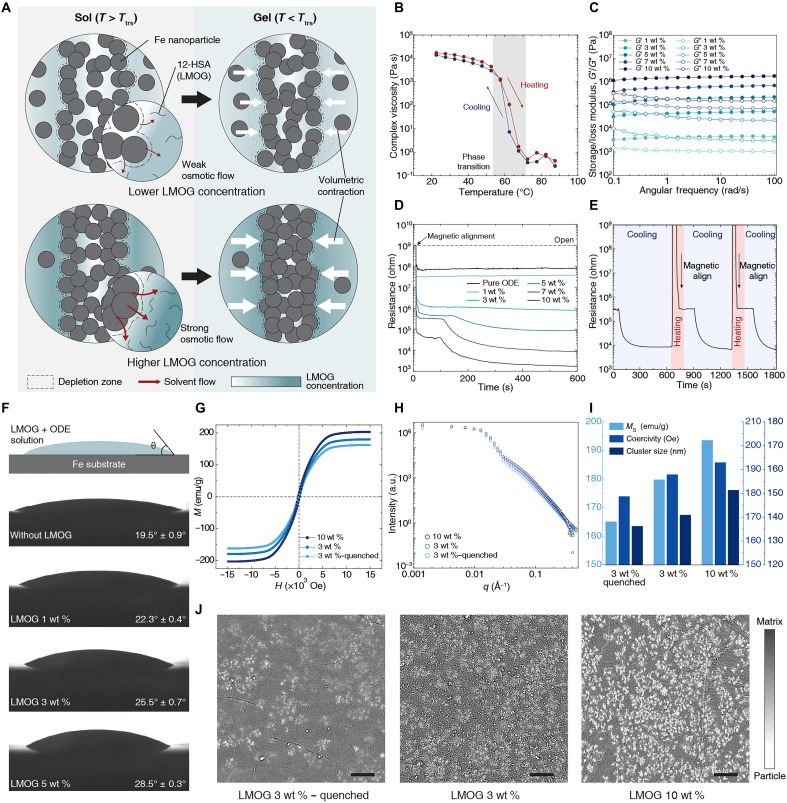
Effects of LMOG concentration on the structure and electrical properties of particle percolation. (**A**) Mechanism of enhanced electrical percolation by LMOG concentration during a phase transition. The LMOG concentration influences the quality of the percolation, although the solution contains the same amount of MNPs. (**B**) Complex viscosity of ODE + LMOG (10 wt %) solution during a subsequent heating and cooling process in a single sample. (**C**) Rheological properties of ODE + LMOG solutions with varying LMOG fractions (1, 3, 5, 7, and 10 wt %) at room temperature. Strain (0.1%) is used for the tests. (**D**) Resistance changes of MNP + LMOG + ODE solutions during cooling from 90°C to room temperature. MNP density (*C*_particle_) is fixed at 0.3 g/cm^3^, but LMOG concentration (ϕ_LMOG_) varies from 1, 3, 5, 7, to 10 wt %. (**E**) Change in resistance during repeated heating, aligning, and cooling cycles (*C*_particle_ = 0.3 g/cm^3^, ϕ_LMOG_ = 7 wt %). (**F**) Static contact angle of LMOG + ODE solution on an iron substrate with varying LMOG concentrations at 70°C (just above *T*_trs_). (**G**) Magnetization curve of patterned nanoparticle assemblies (*C*_particle_ = 0.7 g/cm^3^) subjected to different LMOG concentrations and gelation processes. (**H**) Small-angle x-ray scattering (SAXS) analysis of patterned nanoparticle assemblies with different LMOG concentrations and gelation processes (*C*_particle_ = 0.7 g/cm^3^). (**I**) Saturation magnetization (*M*_s_) and magnetic coercivity are obtained from magnetization curves in (G). Cluster size of nanoparticles is calculated from SAXS data in (H) using Guinier model. (**J**) Cryo-FESEM images of nanoparticle percolation (bright) and organogel matrix (dark) in the plane perpendicular to the direction of conduction. As particle percolation forms densely, it backscatters electrons more strongly, thus showing a brighter signal. Scale bars, 5 μm.

We found that, even when the amount of particles per unit volume is equal, the electrical properties of the nanoparticle assembly are notably affected by the LMOG concentration. Representative cases of low and high LMOG concentrations are depicted (Fig. 3A). Each case is separated by its states: sol state above *T*_trs_ and gel state below *T*_trs_. As the LMOG molecules do not adsorb onto Fe nanoparticles, a depletion zone in which the LMOG concentration is lower than that of the bulk solution forms around the nanoparticles. In the process of magnetic alignment, the depletion zones of the particles rapidly overlap, creating a concentration difference of LMOG between inside and outside of the particle assembly. The imbalance of LMOG concentration is greater in the samples with high LMOG concentration, inducing stronger osmosis than in the samples with low LMOG concentration. It causes the solvent within the particle assembly to diffuse out, thereby strengthening the percolation of particles. As gelation begins, volume contraction of the matrix occurs, inducing inhomogeneous deformation in the preformed particle assembly. Consequently, the higher the concentration of LMOG, the more effectively the volumetric deformation enhances particle percolation during the sol-gel transition. We confirmed the structural reversibility through phase transitions by repeated heating and cooling cycles (Fig. 3B) and measured the mechanical properties according to LMOG concentration (Fig. 3C). In addition, the electrical resistance through particle assembly at varying LMOG concentration was recorded over time (Fig. 3D). During the recordings, the particle concentration was kept constant at 0.3 g/cm^3^. The sample was shaped as a disk with a diameter of 5 mm and a thickness of 1 mm. Measurements were conducted from 90°C (*t* = 0 s) to room temperature (*t* = 600 s). As soon as magnetic alignment occurs, the resistance forms a plateau at specific values, which are strongly influenced by both the initial percolation formed by magnetic alignment and the osmotic solvent redistribution driven by local LMOG concentration differences. Shortly thereafter, as the sol-gel transition begins, a sharp decrease in resistance is observed, particularly at higher LMOG concentrations, due to volumetric contraction. In summary, in the sol state, particle junctions are stabilized by osmosis, while during the phase transition, further stabilization is achieved through squeezing caused by contraction force during gelation (figs. S21 and S22). These electrical characteristics can be reproducibly repeated through the sol-gel transitions (Fig. 3E).

To understand the interaction between the LMOG and Fe nanoparticles, we measured the contact angle of LMOG solutions at 70°C with varying concentrations on Fe substrates (Fig. 3F). A higher contact angle was observed for LMOG-rich solutions, suggesting that the interfacial energy between ODE and Fe is lower than that between 12-HSA and Fe (fig. S23). This indicates that ODE preferentially localizes at the Fe-solution interface, which is energetically more favorable than adsorption of 12-HSA. To further support this interpretation, we conducted Fourier transform infrared spectroscopy (fig. S24). The carbonyl (C═O) stretching peak of the carboxylic acid group in 12-HSA, the primary interaction site, showed no detectable shift when mixed with Fe nanoparticles, compared to the spectrum of pure 12-HSA. This result implies negligible interaction between 12-HSA and Fe nanoparticle, supporting the assumption of a negative adsorption relationship (*41*).

In addition, to demonstrate how LMOG concentration affects electrical resistance after the phase transition, we compared three sample groups: a quenched sample with 3 wt % low LMOG concentration (representing sol-state morphology) and slowly cooled samples with 3 wt % low and 10 wt % high LMOG concentration (each representing gel-state morphologies). To compare the relative size of the clusters in each group, we measured the saturation magnetization (*M*_s_) and magnetic coercivity, both of which increase with cluster size, through hysteresis loop measurements (Fig. 3G) (*42*, *43*). When comparing the two parameters, the higher values in the 10 wt % sample than in the 3 wt % sample suggest that the sample of higher LMOG concentrations more effectively forms large particle assemblies. Furthermore, the smaller values in the quenched 3 wt % sample compared to the slowly cooled 3 wt % sample indicate that spontaneous volume contraction during the sol-gel transition enhances particle percolation (Fig. 3I). The sizes of particle clusters assembled in each sample were investigated using small-angle x-ray scattering (SAXS) (Fig. 3H and fig. S25). The intensity was higher in the low-*q* Guinier region (*q* < 0.01), in the order of slowly cooled 10 wt %, slowly cooled 3 wt %, and quenched 3 wt %. Fitting the SAXS data at low-*q* Guinier region (*q* < 0.01) from these samples to the Guinier model, the radius of the assembled clusters was also measured to be larger in the same order (Fig. 3I and see text S2 for details). Cryo–field-emission scanning electron microscope (cryo-FESEM) images of three samples also show the distribution of nanoparticle percolation across the cross section in the direction of conduction (Fig. 3J). A brighter signal indicates a firmer percolation formed between nanoparticles. Although the particle concentrations were equal, the brightest signal from percolation was observed in the 10 wt % LMOG sample. These results can be attributed to both osmosis, due to differences in LMOG concentration in the sol state, and inhomogeneous deformation during the sol-gel transition.

**Fig. 4.**
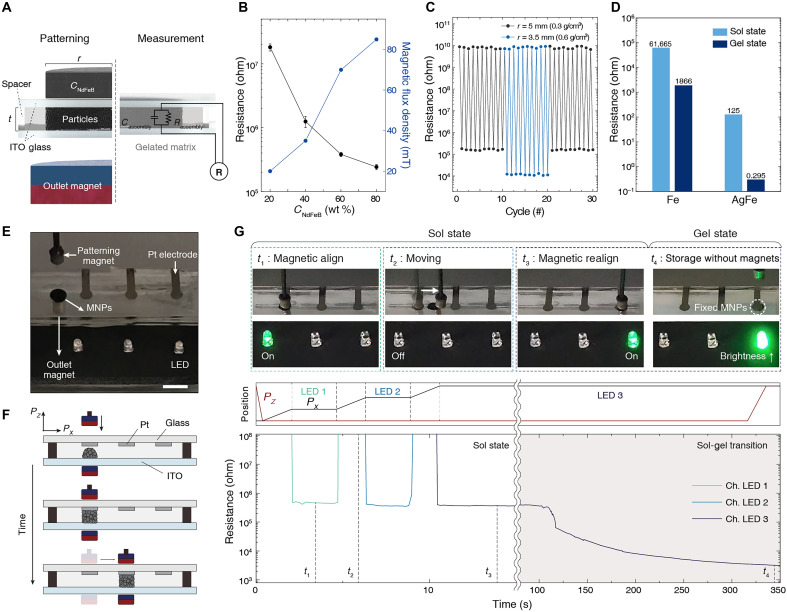
Reconfiguration of an electrical circuit using RAMP. (**A**) A setup for measuring dc resistance and impedance of RAMP after patterning (*t* = 1 mm). (**B**) High concentration of NdFeB particles in the patterning magnet lowers the resistance of RAMP (*r* = 5 mm, *t* = 2 mm, strength of outlet magnet = 200 mT, *C*_particle_ = 0.3 g/cm^3^, ϕ_LMOG_ = 7 wt %). (**C**) Resistance changes of RAMP varying concentration of MNPs during patterning and erasing cycles with fixed amount of particles. After 10 cycles, patterning magnet is switched from *r* = 5 to 3.5 mm (*m*_particle_ = 0.023 g, ϕ_LMOG_ = 7 wt %). (**D**) Resistance changes in the percolation networks composed of Fe and AgFe particles by phase transition (*C*_particle_ = 0.3 g/cm^3^, ϕ_LMOG_ = 7 wt %, *r* = 2.5 mm, *t* = 1 mm). (**E** and **F**) Setup for reconfigurable circuit demonstration with RAMP. Scale bar, 1 cm. An electrical pathway can be formed between Pt electrode and ITO when the magnetic alignment is achieved by moving patterning and outlet magnets. (**G**) Electric resistance of RAMP is recorded during a circuit reconfiguration from LED 1 to LED 3 (*V*_applied_ = 5 V).

After the gelation process with magnetic alignment, a stable conductive network formed by nanoparticle percolation in the organogel was characterized electrically under various conditions (Fig. 4, A to D). The resistance of patterned nanoparticle assembly was measured by varying the strength of patterning magnets with a fixed outlet magnet, which has 200 mT of magnetic flux density (Fig. 4B). The strength of the patterning magnet was determined by the amount of NdFeB particles (*C*_NdFeB_) in the magnets (fig. S6). It was observed that stronger magnetic field from the patterning magnet enhances the interparticle attraction, resulting in lower resistance. The distance between the outlet magnet and the sample has an optimum point for enhancing electrical pathway (fig. S26). The impedance can be used to detect electrical behaviors through particle percolation. As the contact resistance and resistance of the external electrodes are negligible, the inset scheme in Fig. 4A can be interpreted as an equivalent circuit. As the concentration of the conductive unit, iron nanoparticles, increases, electrical percolation forms more effectively, behaving as a more stable resistor across a large range of frequencies (1 Hz to 1 MHz) (fig. S27, A and B). With a fixed amount of iron nanoparticles, we observed changes in impedance by altering the patterning area with external patterning magnets. These impedance changes, with a fixed amount of particles and varying patterning area, are consistently determined by the amount of particles per unit volume (fig. S27C). We observed the impedance change of nanoparticle assembly over time at room temperature (fig. S27D). The impedance of nanoparticle percolation in gel-state matrix is stable for 10 days. We also conducted 30 cycles of repeated patterning and erasing in a single sample to assess its electrical reproducibility across different magnet sizes. The first 10 cycles were performed using a magnet with a radius of 5 mm, followed by 10 cycles using a smaller magnet with a radius of 3.5 mm. The final 10 cycles were then conducted again using the 5-mm-radius magnet. The observed variations in resistance are attributed to differences in particle percolation density (Fig. 4C). Notably, enhanced junction stability due to phase transition of the matrix was observed in percolation network with other materials (Fig. 4D). Fabricated Ag-coated Fe particles (fig. S28), due to their low intrinsic resistance, exhibit relatively low resistance values in both sol and gel states. After the phase transition, enhanced junction stability allows the formation of a highly conductive pathway, with a measured conductivity reaching up to $3.2\times 10^{3}\;S/m$ (fig. S29). This result demonstrates that our RAMP system, by tightening percolation through a phase transitional mechanism, provides a versatile platform applicable across different particle systems, beyond just absolute conductivity performance.

### Reconfigurable electronic demonstration with RAMP

We fabricated a noncontact switching system that could be reconfigurably controlled by an external magnetic field using the RAMP system (movie S4). Pt electrodes were deposited onto the substrate and each electrode was connected to a light-emitting diode (LED) (Fig. 4E and fig. S30). In this reconfigurable noncontact switching demonstration, the patterning magnet is automatically controlled as programmed, and the outlet magnet is configured to be mobile, adjusting to the movements of the patterning magnet (Fig. 4F). Figure 4G shows images of the operation and changes of electrical resistance based on the position of patterning magnet. At the beginning (*t* = 0), the temperature is about 90°C, and the sample was slowly cooled to room temperature. As the magnetic field aligned with the magnets at *t*_1_, an electrical pathway was formed through particle percolation along the *z* direction in the sol state. The resistance records show that low resistance values, which turn on an LED, are achieved only when there is magnetic alignment by both magnets. As the particle percolation is moved by external magnetic field, the circuit becomes electrically open at *t*_2_. At *t*_3_, remotely controlled electrical percolation exhibits reproducible resistance values, enabling stable switching operations. As the temperature of the system decreased, the phase transition from sol to gel occurred at around 100 s. During this transition, inhomogeneous deformation derived from the formation of an organogel network enhanced junction stability between nanoparticles, leading to a decrease in electrical resistance by more than 100 times at *t*_4_.

**Fig. 5.**
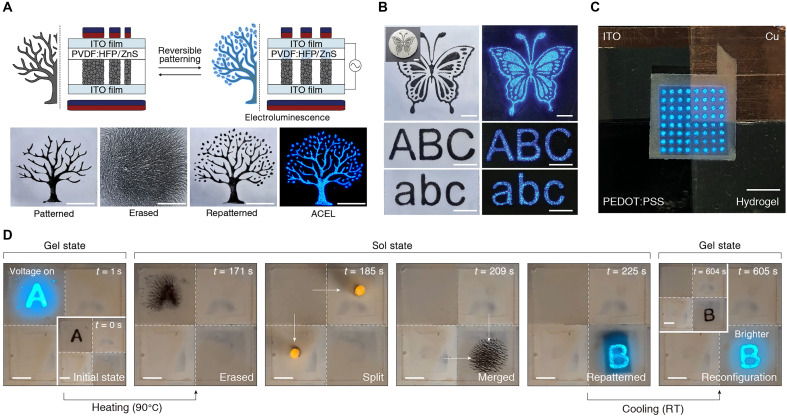
Operation of an ACEL display with RAMP. (**A**) Schematic of reconfigurable ACEL display with RAMP and images of a reconfigurable ACEL sample. Scale bars, 1 cm. (**B**) High-resolution electrode patterning for ACEL display with RAMP (*V*_applied_ = 390 V, frequency = 10 kHz). Scale bars, 5 mm. The inset shows the patterning magnet. (**C**) A dot-patterned electrode with RAMP functions with various counter electrodes (ITO, Cu, PEDOT:PSS, and hydrogel) (*V*_applied_ = 1 kV, frequency = 10 kHz). Scale bar, 1 cm. (**D**) ACEL operation during in situ reconfiguration of RAMP. Scale bars, 5 mm. RT, room temperature.

To demonstrate the applicable use of the RAMP system for reconfigurable electronics, we presented an ACEL display that can be operated in real time, modified multiple times, and stably retain the desired luminescent patterns. The representative setups and processes for a reconfigurable ACEL are demonstrated in Fig. 5A. The luminescent layer, composed of poly(vinylidene fluoride-*co*-hexafluoropropylene) (PVDF-HFP) and ZnS microparticles, is coated on the upper ITO film substrate. By applying an external magnetic field, the assembled nanoparticles, functioning as a patterned electrode, can be reconfigured (movie S5). When alternating voltage is applied to the ITO films, an alternating electric field forms across the patterned electrode, causing electroluminescence in those domains. The RAMP system enables high-resolution patterning of electrodes for electroluminescence, allowing for complex designs and text (Fig. 5B). The fabricated electroconductive patterning is self-standing and flexible, attributed to the mechanical properties of the soft organogel matrix (fig. S31 and movie S6). Since the fabricated patterned electrode is used in contact with external electrodes, it was confirmed that the electrode conformally contacts various materials commonly used for electrodes, such as ITO, copper, poly(3,4-ethylenedioxythiphene)-poly(styrenesulfonate) (PEDOT:PSS), and hydrogel (Fig. 5C). This compatibility, without contact issues, could effectively support its use not only in reconfigurable ACEL displays but also in reconfigurable electronics across various fields. In situ demonstrations of a reconfigurable ACEL display using RAMP in both sol and gel states were performed with an outlet electromagnet (Fig. 5D and movie S7). In the initial state, a patterned character, “A,” is stored within the second quadrant of the organogel matrix, and when voltage is applied, electroluminescence occurs according to the pattern without any external magnetic field. When the matrix transitions to a sol state through heating, the patterned nanoparticle percolation collapses and disperses within the matrix. Applying two different patterning magnetic sources, nanoparticles are split into two small circles. Through magnetic alignment with an electromagnet, these two circles are precisely patterned and emit light as patterned in sol state. Subsequently, the scattered nanoparticles are merged through an external magnetic field, and a different character, “B,” is repatterned in the fourth quadrant of the matrix, distinct from the initial state. By cooling the matrix to a gel state, the reconfigured nanoparticle percolation pattern is stored without an external magnetic field and shows brighter luminescence. Using the RAMP system, we have developed an ACEL display that is reconfigurable in real time, with nanoparticle assemblies functioning as electrodes for electrical and optical signals.

## DISCUSSION

We have proposed a RAMP system for constructing electrically conductive percolation, which features facile controllability and robust junction stability. Our system ensures not only high reconfigurability by introducing nanoscale particles as conductive building blocks but also robust junction stability with a phase transitional matrix. To enhance the controllability over nanoparticles, the magnetic field was precisely tailored through magnetic alignment with outlet magnet. In addition, a soft and insulative phase transitional matrix facilitates reconfigurability with enhanced junction stability of conductive assemblies (table S1). Using the RAMP system, we showed electrical circuits and ACEL displays that are dynamically reconfigured on demand within a single device. By adapting to diverse requirements through simple alternations, the RAMP system can offer customized multifunctional devices suited to individual preferences. Further, by modulating the combination of soft materials and nanomaterials, the RAMP system is expected to be applicable across various electronics such as stretchable electronics, soft robotics, and wearable electronics.

## MATERIALS AND METHODS

### Materials

Fe nanoparticles (US1101) were purchased from US Research Nanomaterials Inc. 12-HSA (TCI Co., H0308) and ODE (TCI Co., O0008) were used for sol-gel transition organogel matrix. Fe foil (Sigma-Aldrich, 356808) was used as a substrate to measure the contact angle with LMOG solution. ITO film (Sigma-Aldrich, 639303) was used for external electrode. NdFeB microparticles (Magnequench, MQFP 15-7) and an elastomer based on silicone (Smooth-On Inc., EcoFlex 0020) were used to fabricate patterning magnets. ZnS microparticles (GG13, GGS42) are purchased from Lonco Company Ltd. Ag-coated Fe particles were made by a ball milling method.

### Fabrication of compliant patterning magnets

To fabricate compliant patterning magnets, an elastomer (EcoFlex, 0020) was prepared by mixing part A and part B in a 1:1 weight ratio. NdFeB microparticles were added into the mixture at concentrations of 20, 40, 60, and 80 wt %. After the mixing process using a mixer (Thinky, ARE-310), the mixture was poured into a 5-mm-thick acrylic mold, designed using a laser cutter (Universal Laser System, VLS3.50). The precursor in the mold was then cured at 60°C for an hour. To transform the mixture into a patterning magnet, the domains inside the NdFeB were aligned with the magnetic field from a magnetizer (ASC Scientific, IM-10-30).

### Preparation of electrode system with RAMP

An LMOG solution was prepared by mixing 12-HSA with ODE at various concentrations (1, 3, 5, 7, and 10 wt %) at 70°C. Conductive ITO glass substrates were used as both the top and bottom electrodes. To form a mold, a 2-mm-thick acrylic spacer was adhered to the bottom ITO substrate using VHB tape, creating a shallow cavity. This cavity was then filled with a heterogeneous mixture of Fe nanoparticles and LMOG-ODE solution. The mold was placed on a hot plate and heated to 90°C until thermal equilibrium was reached. The top ITO substrate was then sealed over the molds using a VHB tape. To pattern the conductive domain by aligning nanoparticles inside the device, the outlet magnet was positioned 1 cm below the device, while the patterning magnet was placed directly above the top ITO glass.

### Preparation of switching and ACEL demonstration with RAMP

To prepare the switching demonstration, a 2-mm-thick acrylic spacer was attached to the bottom ITO glass substrate. Fe nanoparticles and LMOG solution were poured into the mold. The mold was then covered with slide glass, on which patterned Pt electrode had been deposited using a mask (Cressington Scientific Instruments Ltd., 108auto). LEDs were connected to each Pt electrode, respectively, and a voltage supply (Agilent, 33612A) via wiring. The fabricated device was subsequently mounted on a moving stage (Musashi, SM-300SX), and its *z*-axis jaw was replaced with a patterning magnet. For the ACEL demonstration, the bottom substrate and the spacer were prepared in the same manner as for the switching demonstration. An ITO film was used for the top substrate. For the electroluminescence, a homogeneous solution was prepared by mixing electroluminescent particles (pure ZnS for blue and ZnS:Mn for orange), PVDF-HFP, and acetone in a weight ratio of 1:2:10. It was then deposited on the ITO film by dip coating.

### Characterization and measurement

The amplitude of magnetic flux density from fabricated patterning magnets was measured using a gauss meter (Lutron, MG-3002). The probe of gauss meter was positioned just above the surface of the patterning magnet. The rheological properties for organogel containing LMOG were measured using a rheometer (TA Instruments, DHR-2). The complex viscosity of organogel with 10 wt % LMOG was measured during heating and cooling (20° to 100°C; soak time, 5 min). Storage modulus and loss modulus were measured as a function of angular frequency (ranging from 0.1 to 100 rad/s at a fixed strain of 0.1) with varying LMOG concentrations (1, 3, 5, 7, and 10 wt %). To determine the sol-gel transition temperature of the organogel, DSC analysis (TA Instruments, Discovery DSC 2500) was performed (20° to 100°C, 10°C/min). Contact angles between 3-μl drops of LMOG solution (0, 1, 3, and 5 wt %) and an iron substrate maintained at 70°C were measured using a contact angle measurement (FemtoFAB, Smart Drop). Magnetic hysteresis loops of MNPs and their assemblies in organogel with varying LMOG concentrations were measured using a vibrating sample magnetometer (Lake Shore, VSM-7410). The size of the nanoparticle cluster was determined using SAXS (Xenocs, XEUSS 2.0) and calculated with Guinier fitting model. To observe the effect of LMOG concentration on the assembly morphology of nanoparticles, samples with different LMOG concentrations were prepared. To compare morphology of assembly between the sol and gel states, one sample was quenched with liquid nitrogen in the sol state, while others were allowed to cool naturally. To observe the particle percolation structure, the sample was analyzed using x-ray microscope (Xradia 620 Versa). For SEM imaging, the nanoparticle assembly in the organogel was freeze dried (Mareuda, MLB-9003), and its morphology was observed using SEM (Carl Zeiss, Sigma-Aldrich). To minimize damage to the morphology of nanoparticle assembly during solvent removal, the image was also observed using cryo-FESEM (FEI, Talos L120C). Electrical signals from various experimental setups were measured by connecting ITO electrodes of the fabricated device using a multimeter (Agilent, 34461A) and an electrochemical analyzer (Gamry, Reference 600+).

## Supplementary Materials

### The PDF file includes:

Supplementary Text S1 to S3 Figs. S1 to S31 Table S1 Legends for movies S1 to S7 References
