# Supplementary material for: Dynamically assembled magnetic nanoparticles in a phase transitional matrix for reconfigurable electronics
Source: Sci Adv. 2025 Sep 12;11(37):eadw6611. doi: 10.1126/sciadv.adw6611 (PMC12429006; doi:10.1126/sciadv.adw6611)
Supplement: Supplementary file 1 — Supplementary Text S1 to S3 Figs. S1 to S31 Table S1 Legends for movies S1 to S7 References [file sciadv.adw6611_sm.pdf]

Science Advances
AAAS

# Supplementary Materials for

## Dynamically assembled magnetic nanoparticles in a phase transitional matrix for reconfigurable electronics

Min-Gyu Lee *et al.*

Corresponding author: Jeong-Yun Sun, jysun@snu.ac.kr

*Sci. Adv.* **11**, eadw6611 (2025)
DOI: 10.1126/sciadv.adw6611

**The PDF file includes:**

Supplementary Text S1 to S3
Figs. S1 to S31
Table S1
Legends for movies S1 to S7
References

**Other Supplementary Material for this manuscript includes the following:**

Movies S1 to S7

## SUPPLEMENTARY TEXT

### Supplementary Text 1. Finite Element Method (FEM) simulation

For the in-depth understanding of the magnetic properties of the reconfigurable electrode presented in Fig. 1-2, fig. S8, 9, and 13, computational simulations were performed by using COMSOL Multiphysics. Magnetic fields module, particle tracing for fluid flow module, and laminar flow module were used to observe the assembly of the magnetic particles with and without magnets. To observe the magnetic field and magnetic flux density of the system, only magnetic fields module was used. For all simulations, the following input parameters were used:
Due to the constraints of computational resources, the number of particles were limited to 500 and the diameter is set to 160 μm. In the real system, about 0.02 g of Fe magnetic particles with diameter of 40 nm and density of 7.9 g/cm$^3$ were used. Therefore, 1 magnetic particle weighs $5.05 \times 10^{-16} g$ and $3.96 \times 10^{13}$ particles are in the sample. To reduce the number of particles to 500 in the simulation, particles must be enlarged about 4294 times bigger than the real system to compensate the total volume. In our simulation, to make the number simple, the diameter of the magnetic particle is assumed to be 4,000 times larger than the real system. Additionally, the magnetic flux densities of the magnets for the simulations involving magnetic particles were proportionally increased to account for the enlarged particle size.
Particle density = 7.9 g/cm$^3$, collision diameter = 160 μm, interaction strength = 10$^{-3}$ J, and cut-off length of the Lennard-Jones potential is 20 times larger than the particle diameter. For material properties, relative permeability of air = 1.0006, magnet = 1.05 and magnetic particle = 100. For fig S14, 16, and 17, where only the magnetic fields within the system are simulated without particles, magnetic flux densities of 80 mT and 200 mT were used for the patterning and outlet magnets, respectively. Assuming the particle size is enlarged by a factor of 4,000, the magnetic flux densities for the simulations with particles were set as follows: 320 T for the patterning magnet and 800 T for the outlet magnet. For the mechanical properties of the fluid, dynamic viscosity of the fluid = 0.3562 Pa·s, density of the fluid = 789 kg/m$^3$.
The detailed information about the simulation conditions in this study is provided below.

1. Governing equation
   1) Magnetic flux conservation (To define the magnetic field)

$$\nabla \cdot B = 0, \quad B = \mu_0 \mu_r H, \quad H = -\nabla V_m \qquad [1]$$

   - B : Magnetic flux density
   - $\mu_0$ : Vacuum permeability
   - $\mu_r$ : Fluid relative permeability
   - $H$ : Magnetic field strength
   - $V_m$ : Magnetic scalar potential

   2) Magnetophoretic force equation (The particle-magnetic field interaction)

$$F_m = 2\pi r_p^3 \mu_0 \mu_r K \nabla |H|^2, \qquad K = \frac{\mu_{r,p} - \mu_r}{\mu_{r,p} - 2\mu_r} \qquad [2]$$

- $F_m$ : Magnetophoretic force
- $r_p$ : Radius of the particle

- $\mu_0$ : Vacuum permeability
- $\mu_r$ : Fluid relative permeability
- $H$ : Magnetic field strength
- $\mu_{r,p}$ : Relative permeability of the particle

3) Lennard-Jones equation (The particle-particle interaction)

$$F = \frac{24\varepsilon}{\sigma}\sum_{j=1}^{N}\left[2\left(\frac{\sigma}{|r-r_j|}\right)^{13} - \left(\frac{\sigma}{|r-r_j|}\right)^{7}\right]\left(\frac{r-r_j}{|r-r_j|}\right), \qquad [3]$$

- $r$ : Distance between two atoms,
- $\sigma$ : Collision diameter (= bond length)
- $\varepsilon$ : Interaction strength (= bond energy)

4) Navier-Stokes equation (To describe the motion of viscous fluid substances)

$$\rho(u\cdot\nabla)u = \nabla\cdot[-pI + K] + F,\ \rho\nabla\cdot u = 0,\ K = \mu(\nabla u + (\nabla u)^T) \qquad [4]$$

- $\rho$ : Density of the fluid
- u : Velocity of the fluid
- $p$ : Pressure
- $I$ : The identity tensor
- $\mu$ : Dynamic viscosity of the fluid
- T : Temperature

5) Stokes' law (To describe the drag force between the particle and the fluid)

$$F_D = \frac{1}{\tau_p}m_p(u-v), \tau_p = \frac{\rho_P d_P^2}{18\mu},\ F_{vm} = \frac{1}{2}m_f\frac{d(u-v)}{dt}, m_f = \frac{1}{6}\pi d_p^3\rho,$$

$$\frac{d(u-v)}{dt} = \frac{\partial(u-v)}{\partial t} + v\cdot\nabla(u-v),\ F_p = m_f\frac{Du}{Dt}, \frac{Du}{Dt} = \frac{\partial u}{\partial t} + u\cdot\nabla u \qquad [5]$$

- $F_D$ : The drag force
- $\tau_p$ : Particle velocity response time
- $m_p$ : Mass of the particle
- $u$ : Flow velocity relative to the object
- $v$ : Kinematic viscosity of the fluid
- $\rho_P$ : Density of the particle
- $F_{vm}$ : The virtual mass term
- $m_f$ : The mass of the fluid displaced by the particle volume
- $F_p$ : The pressure gradient force
- $d_p$ : Diameter of the particle
- $\rho$ : Density of the fluid
- $\mu$ : Dynamic viscosity of the fluid
- $t$ : Time

2. Boundary conditions

1) Magnetic insulation at the edge of the background area

$$n\cdot B = 0$$

2) Out-of-plane thickness = 10 mm
3) Wall condition of the particle at the sample edge : Bounce

$$v = v_c \cdot 2(n \cdot v_c)n$$

- $v$ : Velocity of the particle after striking the wall
- $v_c$ : Velocity of the particle when striking the wall

4) Wall condition of the particle at the sample edge below patterning magnet: Freeze

$$v = v_c$$

5) Wall condition of the fluid at the sample edge : No slip

$$u = 0$$

6) Boundary condition of the fluid at the inlet and outlet : Fully developed flow

$$u \cdot t = 0, P_{inl} \ : \ P_{av} = \frac{1}{A}\int_{\partial\Omega_{inl}} pdS \ , A = \int_{\partial\Omega_{inl}} dS$$

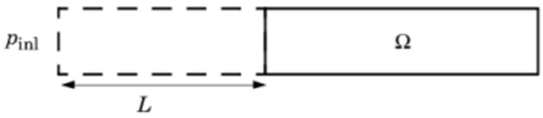


- $P_{inl}$ : Pressure which is applied on the inlet/outlet
- $P_{av}$ : Average pressure of the fluid
- $A$ : Cross-sectional area of inlet/outlet
- $L$ : Length of a straight channel which is a virtual extrusion of the inlet cross section
- $\Omega_{inl}$ : Computational domain of the flow

**Supplementary Text 2. Small angle X-ray scattering analysis**

The percolation of nanoparticles characterization was performed using SAXS measurements (Xenocs, Xeuss 2.0). Scattering vector ($q = 4\pi \cdot \sin\frac{\theta}{\lambda}$, where λ is the X-ray wavelength and $\theta$ is half of the scattering angle) between $1.4 \times 10^{-4} < q < 0.14$ Å$^{-1}$ was detected with wavelength of λ = 1.54189 Å. The analysis of SAXS experimental data was using Guinier fitting model to obtain radius of gyration.

$$I(q) = I(0) \cdot e^{-q^2 R_g^2/3}$$

where $R_g$ is the radius of gyration for cluster, and I(0) is the intensity at zero scattering angle (q = 0). The value of I(0) can be determined by Guinier approximation. The radius ($R_c$) of sphere-like cluster can be written as

$$R_g^2 = \frac{3}{5} \cdot R_c^2$$

**Supplementary Text 3. Calculating the time scale of ODE diffusion**

Based on small-angle X-ray scattering (SAXS) data (Fig. 3I), the average cluster size of the nanoparticle assemblies was approximately 150 nm. Using the experimentally measured complex viscosity at 87.52 °C (0.2598 Pa·s), extracted from Fig. 3B, and applying the Stokes–Einstein

equation, we calculated a diffusion coefficient of ODE in composite to be approximately $D = 1.12 \times 10^{-12} m^2/s$.

The diffusion coefficient was calculated using the Stokes-Einstein equation:

$$D = \frac{k_B T}{6\pi\eta r},$$

where $k_B$ is the Boltzmann constant ($1.38 \times 10^{-23} J/K$),

T is the absolute temperature (87.52 °C = 360.67 K),

$\eta$ is the dynamic viscosity of the medium (0.2598 Pa·s),

and $r$ is the hydrodynamic radius of ODE, approximately as 0.9 nm ($9 \times 10^{-10}\ m$) by assuming a spherical geometry based on its molecular volume.

Using this diffusion coefficient, we estimated the time required for molecular diffusion across a distance of 150 nm—comparable to the observed cluster size—based on the root-mean-square displacement formula for the diffusion:

$$rms\ displacement \approx \sqrt{D}t,$$

Rearranging this equation gives:

$$t \approx \frac{(rms\ displacement)^2}{D},$$

Substituting the values (distance = 150 nm, $D = 1.12 \times 10^{-12} m^2/s$ ), we obtain:

$$t \approx \frac{(150 \times 10^{-9}\ m)^2}{1.12 \times 10^{-12}\ m^2/s} \approx 0.02\ s$$

This estimation is based on random diffusion of ODE and does not account for osmotic flow or active transport. Still, the timescale—on the order of tens of milliseconds—indicates that solvent redistribution over ~150 nm can occur fast enough to match the initial resistance drop observed after magnetic alignment. This supports the plausibility of our interpretation that LMOG exclusion leads to local concentration gradients, which in turn drive rapid solvent migration in the sol state.

**SUPPLEMENTARY FIGURE**

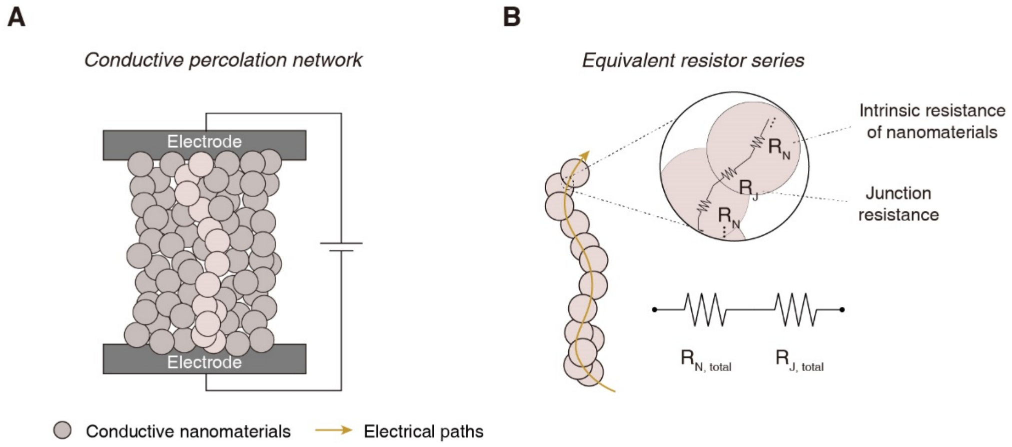


**Fig. S1. Schematic illustration of the conductive pecolation network and its equivalent circuit.** (**A**) The percolation network composed of nanomaterials connected to two electrodes. (**B**) A linear percolation of nanomaterials forms an electrical path (yellow arrow). This path consists of two types of resistance: the intrinsic resistance of nanomaterials, $R_N$, and the junction resistance, $R_J$, arising from the contacts between them. In the equivalent circuit, the total resistance of the nanomateial network is represented as a series combination of two resistors, $R_{N,\ total}$ and $R_{J,\ total}$ (*19*).

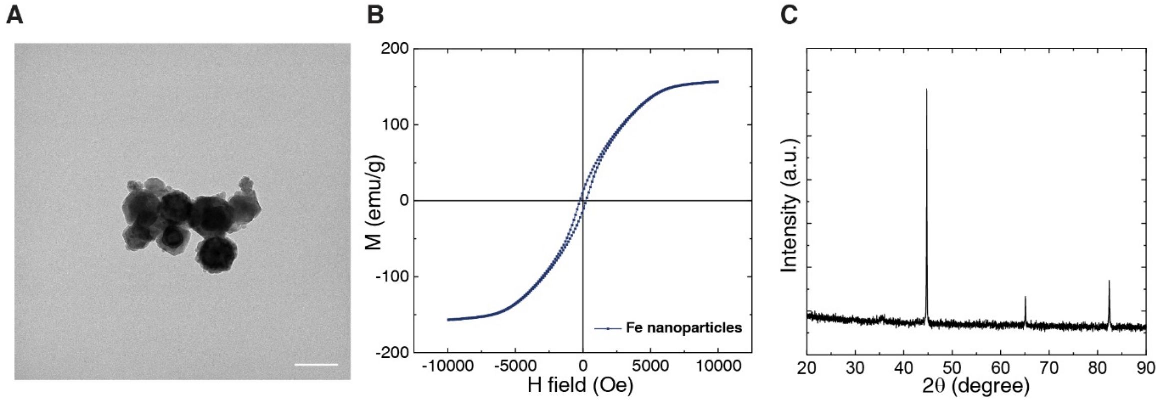


**Fig. S2. Fe nanoparticle characterization.** (**A**) TEM image of Fe nanoparticles (scale bar: 100 nm). (**B**) Magnetic hysteresis loop of Fe nanoparticles. (**C**) XRD peaks of Fe nanoparticles.

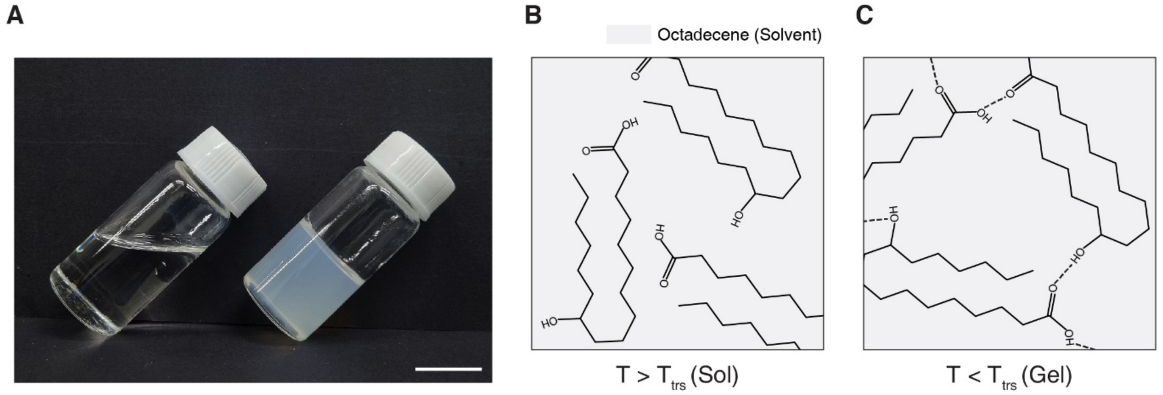


**Fig. S3. Sol-gel transition of organogels.** (**A**) Photograph of sol-gel transition of organogel with octadecene and LMOG (scale bar: 2 cm). (**B**) Above the $T_{trs}$, increased thermal energy disrupts hydrogen bonds between 12-HSA molecules, preventing the formation of a stable network necessary for gelation with octadecene, thus remaining as sol-state. (**C**) 12-HSA forms hydrogen bonds through its hydroxyl and carboxyl groups. These interactions enable 12-HSA molecules to self-assemble below the $T_{trs}$, resulting in the formation of an organogel with octadecene.

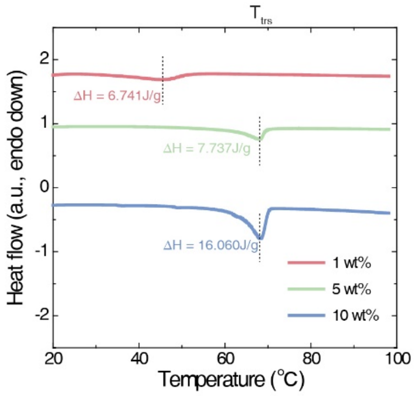


**Fig. S4. DSC data for organogel with varying LMOG concentrations (1, 5, and 10 wt%).** With increasing LMOG concentration, the $T_{trs}$ remains nearly unchanged, while the transition requires more energy due to an increase in hydrogen bonds between LMOGs.

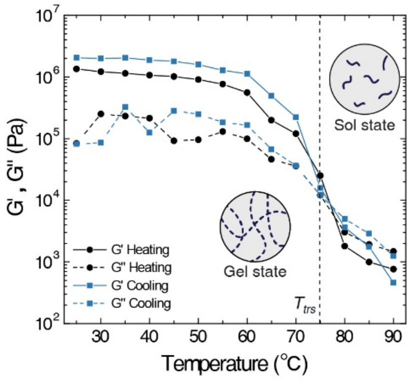


**Fig. S5. Storage and loss modulus of the organogel as a function of temperature during heating and cooling. (from 20 °C to 90 °C) .** At the $T_{trs}$, an inversion between the storage modulus and the loss modulus was observed. After repeated heating and cooling cycles, its modulus trend remains at a similar level.

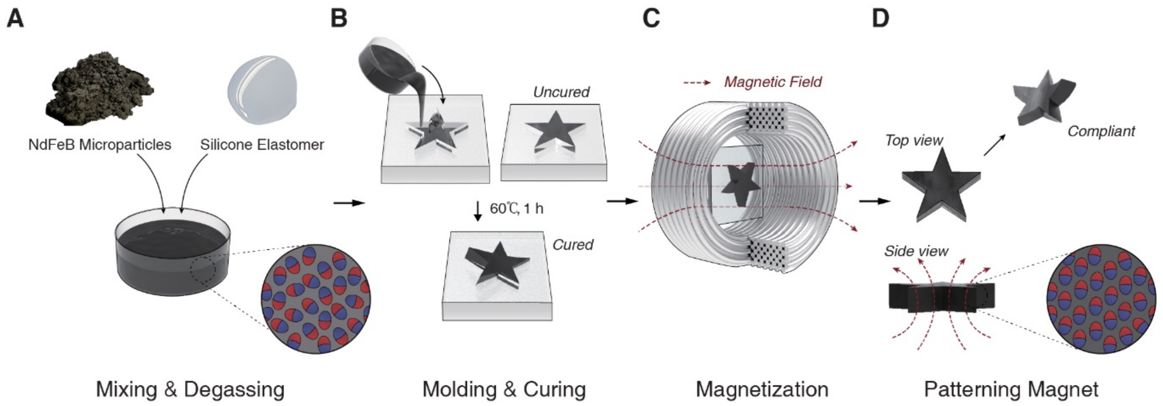


**Fig. S6. Fabrication of compliant patterning magnets.** (**A**) Uniformly blend NdFeB microparticles with silicone elastomer precursor using a mixer and conduct degassing process for the removal of gas bubbles. (**B**) The homogeneously mixed precursor was poured into a mold made by 3-D printing, and cured in a 60 °C oven for an hour. In this state, magnetic moments of the particles in the elastomer are radomly aligned. (**C**) To transform the sample into a magnet, it is placed onto a substrate and subjected to intantaneous strong magnetic field (~2.7 T) from a magnetizer. (**D**) The completed magnet exhibits compliance while having magnetic moments aligned in one direction, allowing it to serve its role as a patterned magnet.

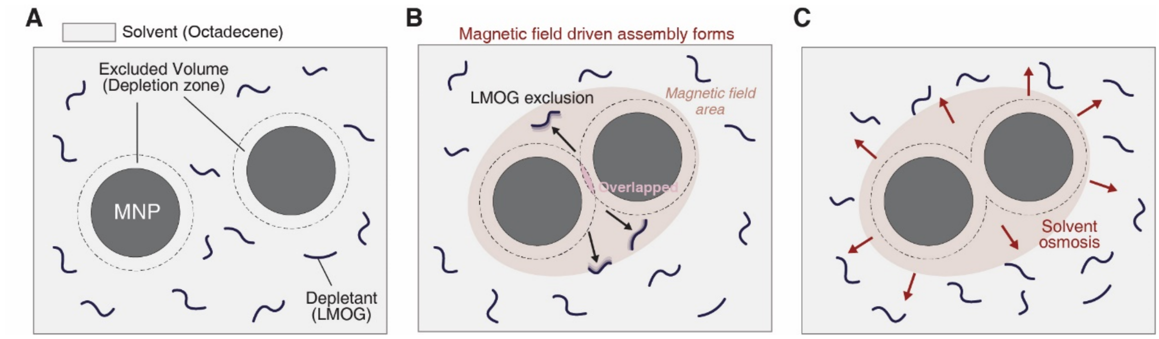


**Fig. S7. The influence of depletant (LMOG) on the particle percolation network in the sol-state.** (**A**) In the vicinity of magnetic nanoparticles (MNPs), an excluded volume, referred to as the depletion zone, forms due to steric hindrance, which restricts the occupancy of the depletant (*33*). (**B**) When a magnetic field is applied, the interactions between MNPs increase, causing the excluded volumes to overlap. (**C**) This leads to an uneven distribution of local mean osmotic gradient (LMOG) concentrations, thereby causing osmosis, where the solvent (ODE) diffuses out from the particle percolation (*36*).

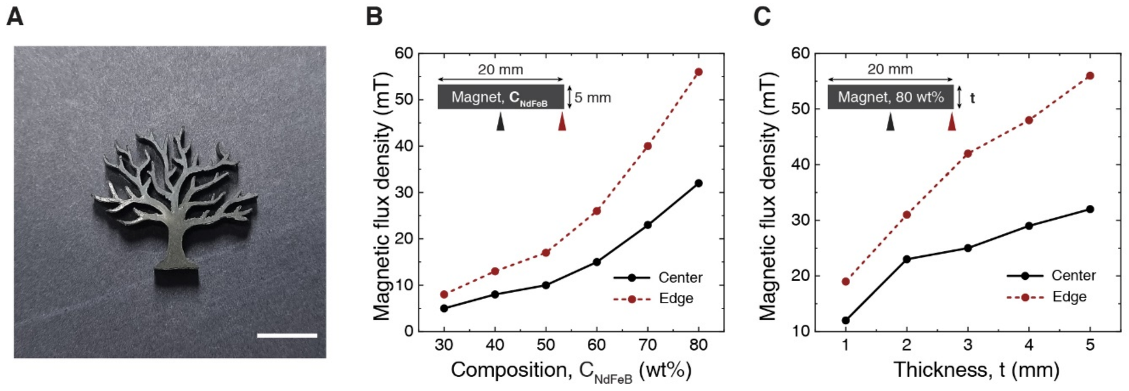


**Fig. S8. Magnetic field strength of the fabricated pattering magnet.** (**A**) Photograph of fabricated a patterning magnet (tree shape, scale bar : 10 mm). (**B**) The magnetic field strength at both the center and edge of the magnet increases with the addition of NdFeB particles, due to enhanced magnetic domain alignment and magnetic saturation, which collectively amplify the overall field strength. (**C**) The magnetic field strength also increases with the thickness of the magnet. ($C_{NdFeB}$ = 80 wt%)

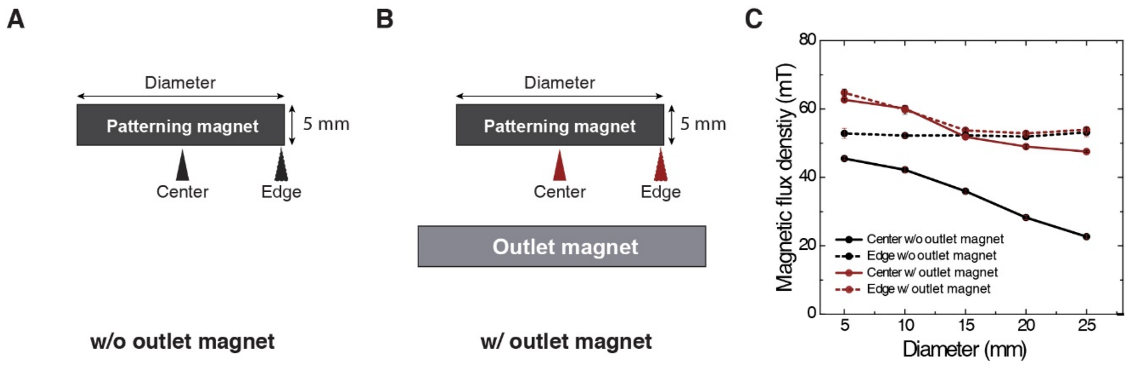


**Fig. S9. Magnetic field strength of patterning magnet with the adoption of an outlet magnet.** Schematic illustration of the magnet arrangement without (**A**) and with (**B**) outlet magnet. (**C**) In the absence of an outlet magnet, the magnetic flux density at the center decreases as the magnetic field disperses outward. This effect becomes more pronounced as the diameter of the patterning magnet increases. However, with the presence of an outlet magnet, the magnetic field becomes more aligned, reducing the imbalance in magnetic flux density between the center and the edge.

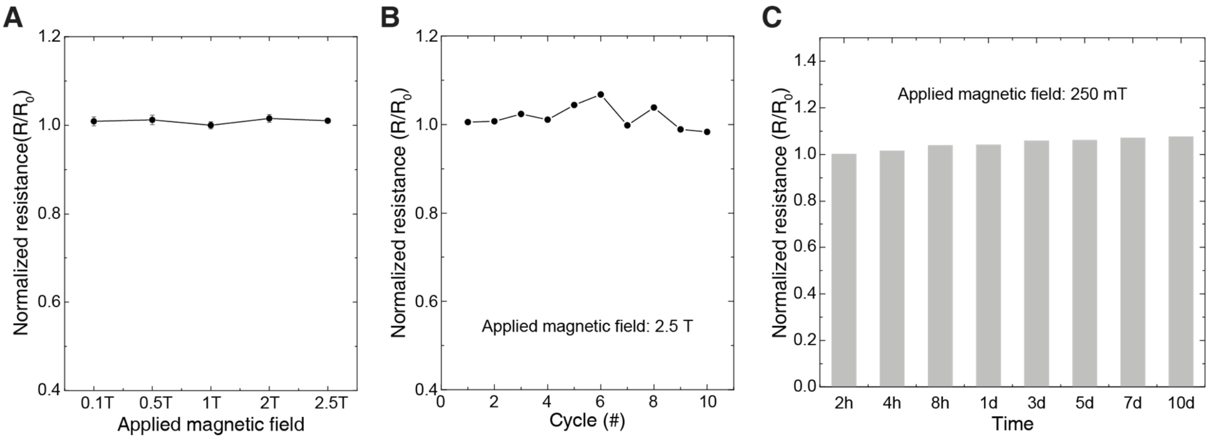


**Fig. S10. Resistance changes of patterned percolation within the organogel ($C_{LMOG}$: 10 wt%).** (**A**) Resistance values normalized to the initial resistance ($R_0$) after exposure to magnetic fields of varying strengths. The percolation network within the organogel remains stable under magnetic fields up to 2.5 T. (**B**) Resistance change upon repeated exposure to a 2.5 T magnetic field. Even after ten cycles of magnetic exposure on a single sample, the resistance change is negligible, confirming the robustness of the percolation network. (**C**) Resistance change of the patterned electrode under continuous exposure to an external magnetic field of 250 mT

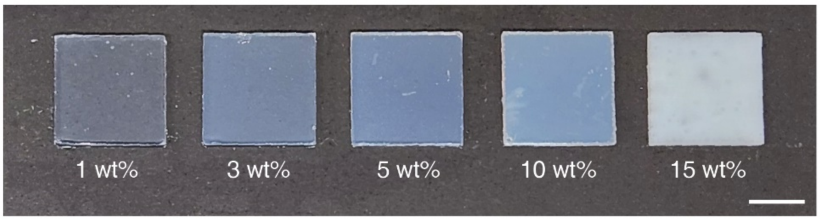


**Fig. S11. The opacity of organogel with various LMOG concentration (1, 3, 5, 10, 15 wt%, scale bar: 1cm).**

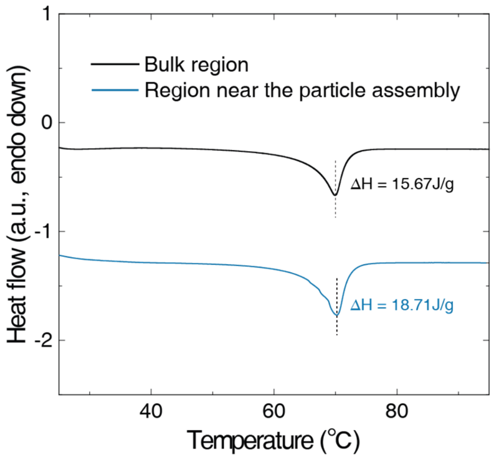


**Fig. S12. DSC data collected from the region near the particle assembly and from the bulk region.** The organogel adjacent to the particle assembly exhibited a higher enthalpy for the phase transition, suggesting that a denser gel network was formed due to the accumulation of LMOG expelled by the depletion effect ($C_{LMOG}$ = 10 wt%).

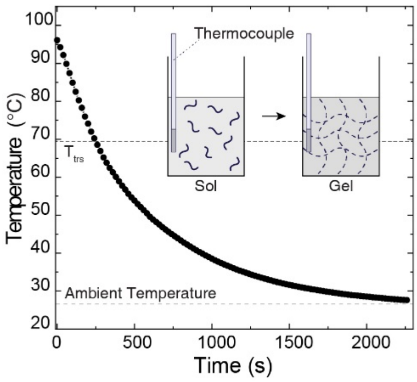


**Fig. S13. The temperature change of the matrix during the sol-gel transition from 90°C to 25°C.** As the latent heat of sol-gel transition is low compared to that of solid-liquid transition, no significant effect on the temperature gradient is observed.

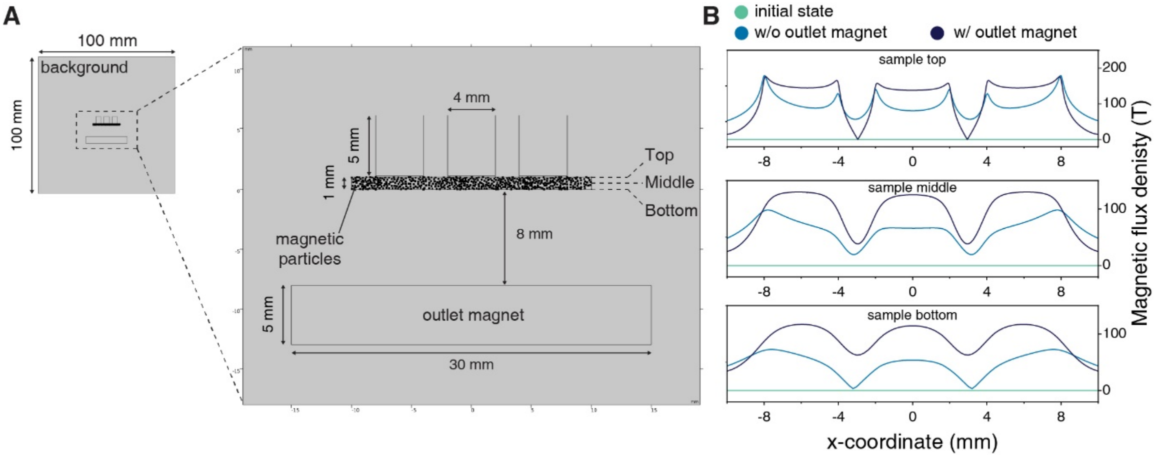


**Fig. S14. 2-D computational simulation for the behaviors of magnetic particles under applied magnetic field with and without outlet magnet.** (**A**) Geometrical settings for the simulation. Detailed settings are written in Supplementary Text 1. (**B**) Magnetic flux density at the sample top, middle, and bottom, respectively.

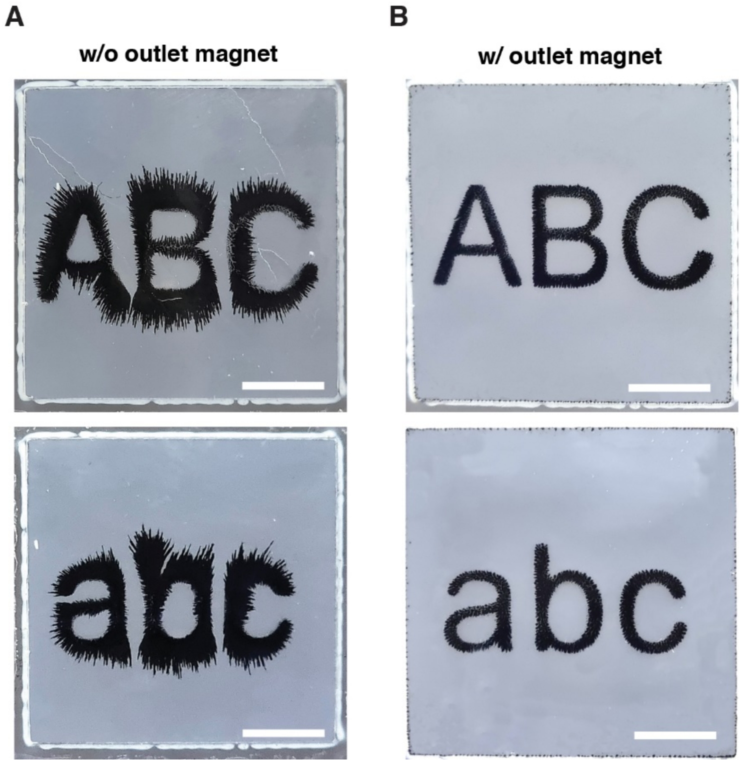


**Fig. S15. Photographs of the character patterning.** Magnetic particle assembly-based characters (ABC, abc) are patterned without (**A**) and with (**B**) the outlet magnet. (scale bar: 5 mm)

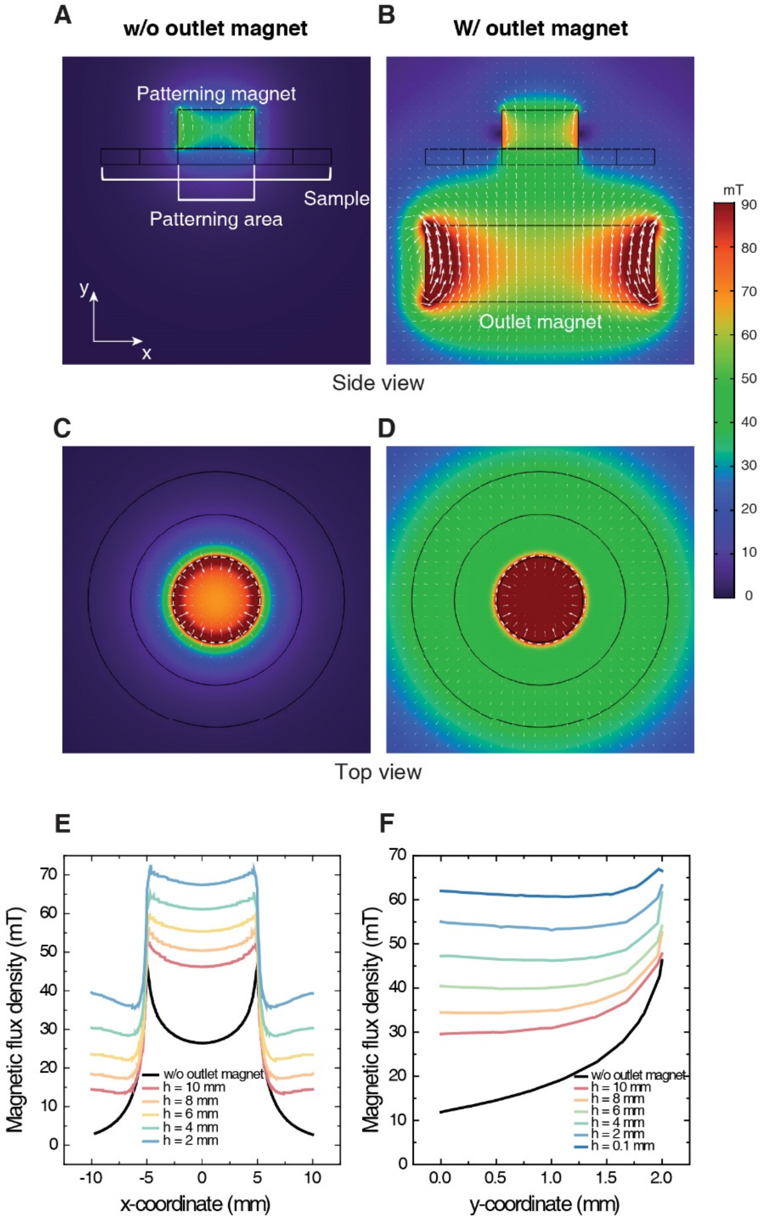


**Fig. S16. 3-D magnetic field simulation results with and without cylindrical outlet magnet.** Only with the patterning magnet, magnetic field is dispersed in every direction and showed edge effect near top of the sample (**A** and **C**). On the contrary, by introducing outlet magnet at the bottom of the sample, magnetic field is aligned with minimal edge effect (**B** and **D**). Magnetic flux density graphs by x-coordinate and y-coordinate (**E** and **F**) show enhanced alignment and reduced edge effect when the outlet magnet is approaching to the bottom of the sample.

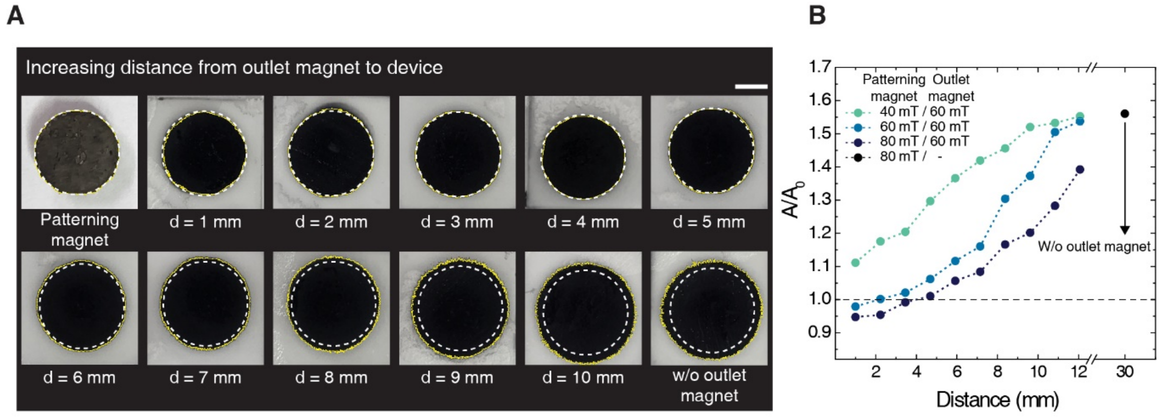


**Fig. S17. Optimization of conditions for magnetic patterning** (**A**) Photographs of patterned nanoparticle assemblies in the organogel with increasing distance from the outlet magnet to the device (scale bar: 5 mm). When a strong outlet magnet is close to the device, patterns are smaller than the patterning magnet. As the distance is optimized, patterns match the size of the patterning magnet. Beyond the optimal distance, weaker magnetic alignment results in larger patterns. (**B**) Patterning resolution with varying magnetic strengths of the patterning magnet and fixed outlet magnet strength. The optimal outlet magnet distance is determined to achieve high-resolution patterning.

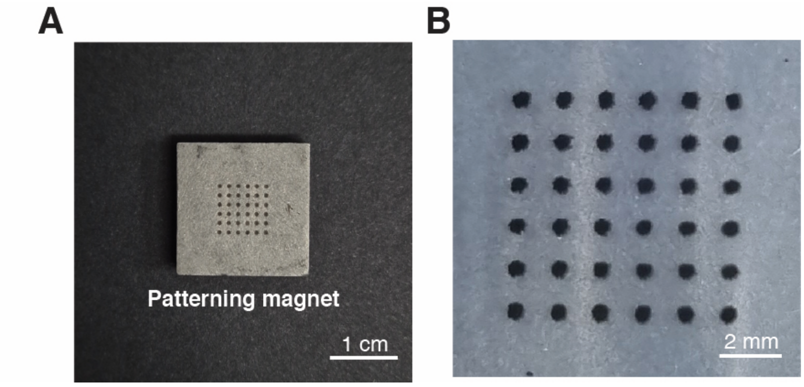


**Fig. S18. Demonstration of the high resolution (~500 µm) electrode patterning based on magnetic alignment.** (**A**) Patterning magnet with a 500 µm dot pattern. (**B**) The patterned particle percolation.

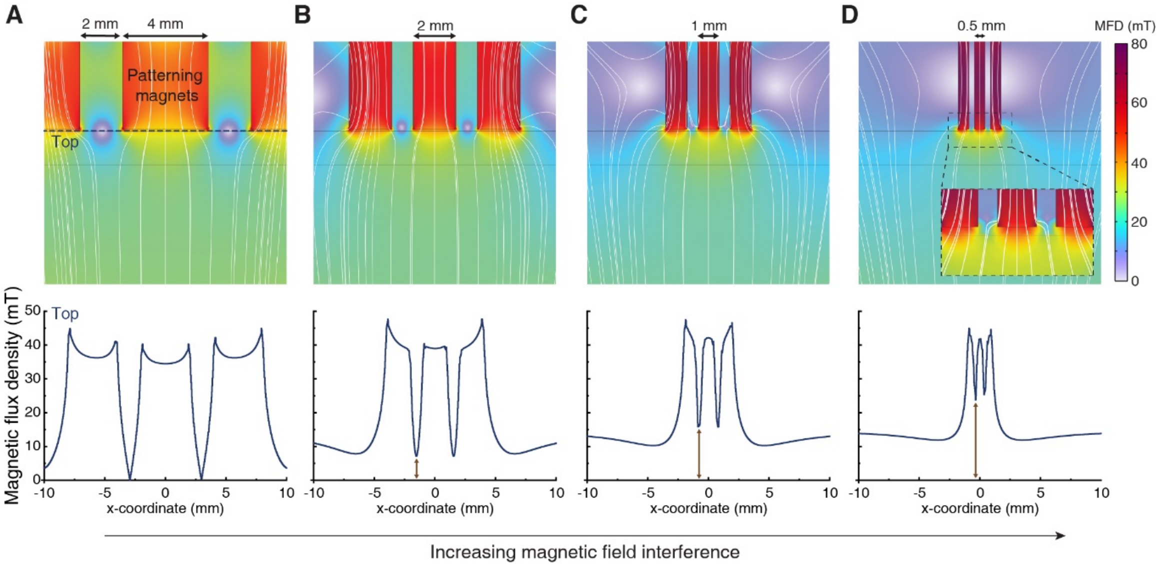


**Fig. S19. 2-D magnetic field simulation of three patterning magnets with different resolution (A** to **D, 4 mm, 2 mm, 1 mm, and 0.5 mm).** An outlet magnet of size 30 mm is placed 9 mm below the patterning magnet. The gap between magnets is half of their length. The more the size and distance decrease, the more the interference of the magnetic fields of the patterning magnets intensifies.

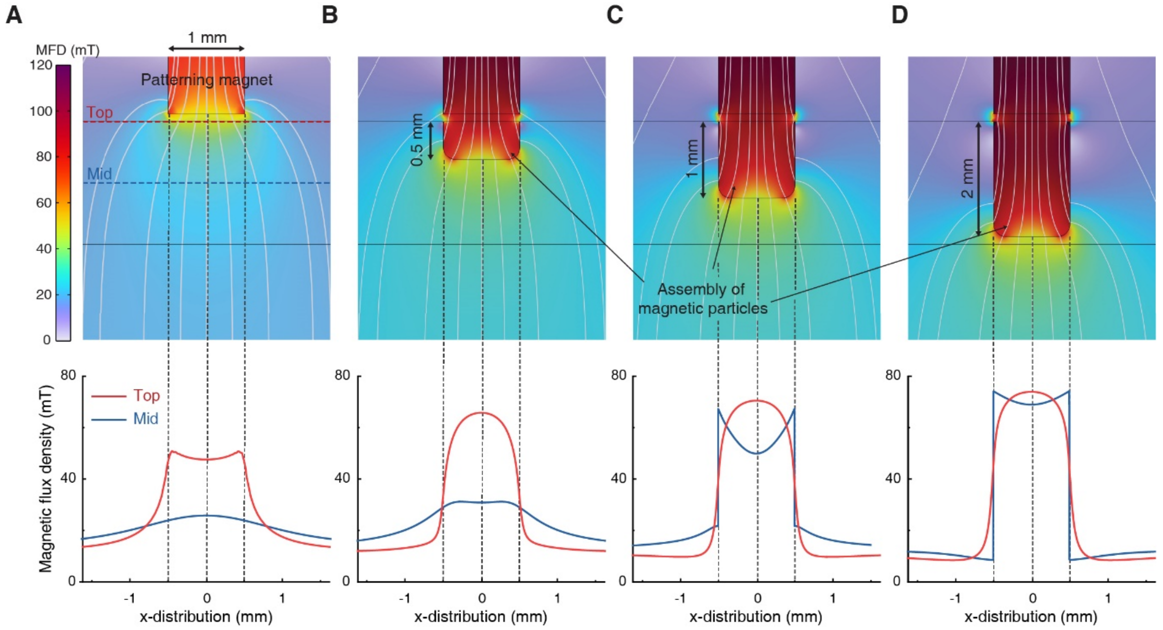


**Fig. S20. 2-D magnetic field simulation with and without the assembly of magnetic particles.** (**A**) Magnetic field distribution with only patterning magnet. Magnetic field distribution with the assembly of magnetic particle of height 0.5 mm (**B**) 1.0 mm (**C**) and 1.5 mm (**D**). Relative permeability of the assembly is 100.

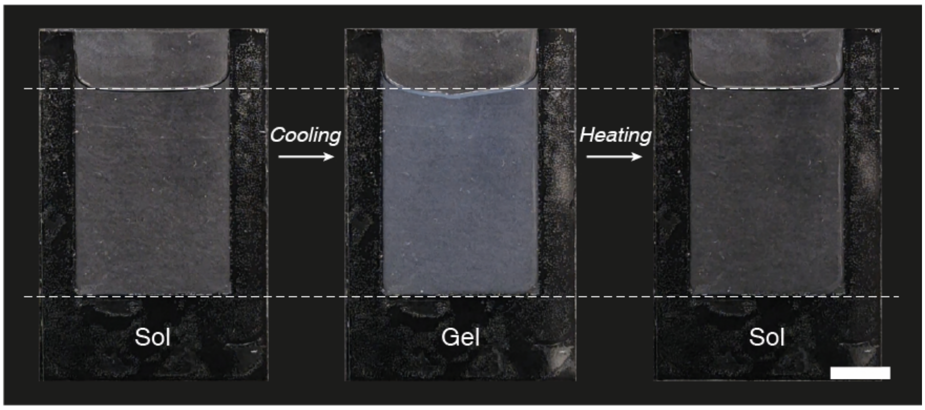


**Fig. S21. Images of the sample during the reversible sol-gel transition, showing the volumes in the sol and gel states.** Image analysis confirmed a volumetric contraction of approximately 1.56% during the transition. ($C_{LMOG}$ = 3 wt%, Scale bar = 5 mm)

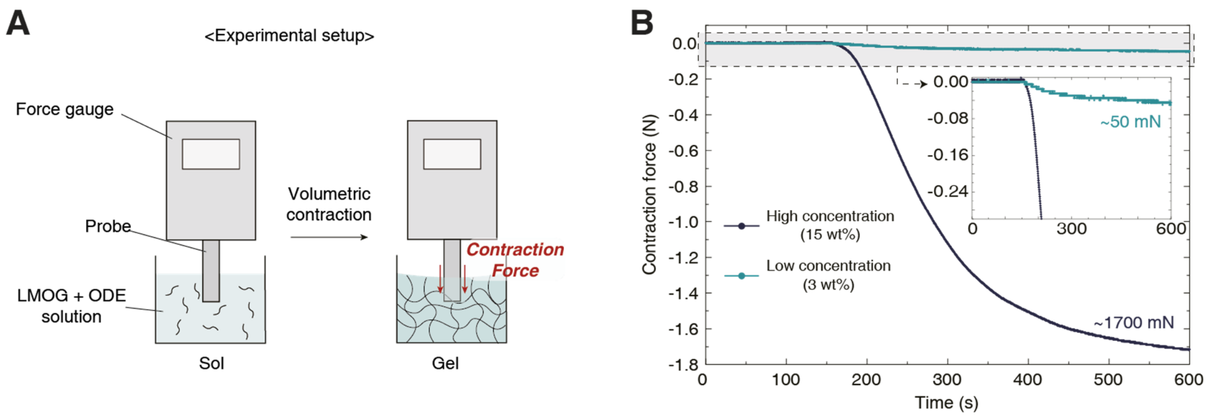


**Fig. S22. Contraction force measured by a force meter during the sol-gel transition.** (**A**) Experimental setup used to measure the contraction force during sol-gel transition. (**B**) Contraction force measured as the sol-gel transition progresses. Higher LMOG concentrations generate greater contraction forces compared to lower concentrations.

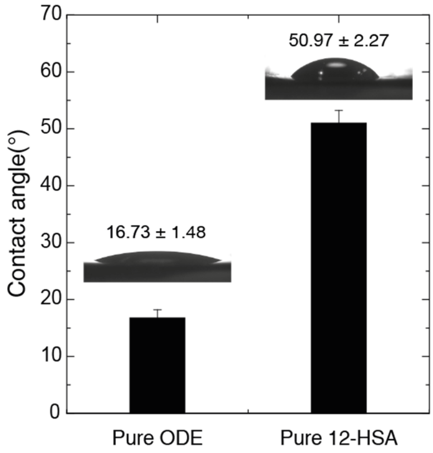


**Fig. S23. Contact angle of pure ODE and 12-HSA with Fe substrate.**

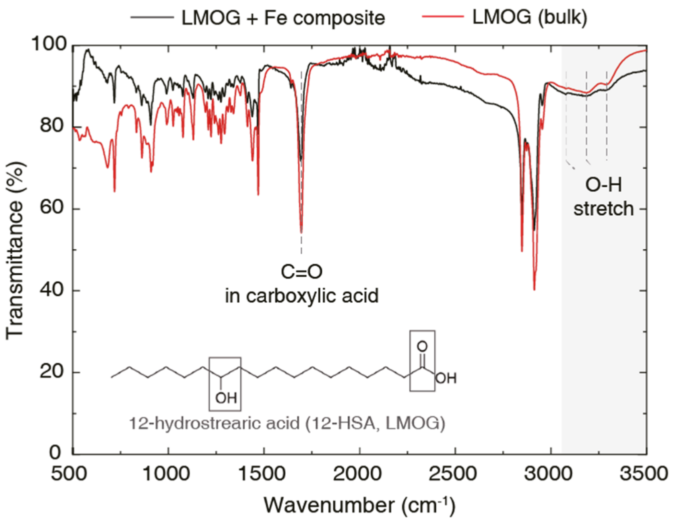


**Fig. S24. FT-IR spectra of pure 12-HSA and 12-HSA mixed with Fe nanoparticles.** The carbonyl (C=O) stretching peak of the carboxylic acid group appears at the same wavenumber in both samples, indicating no detectable shift. This result suggests negligible interaction between 12-HSA and Fe, supporting the presence of a negative adsorption relationship between the LMOG and the particle surface.

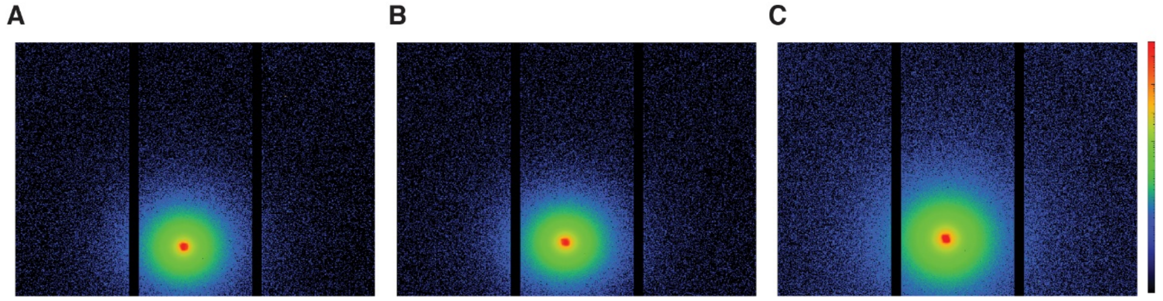


**Fig. S25. SAXS 2-D scattering patterns of nanoparticle assembly in oranogel with the different LMOG concentrations and the gelation process.** As the aggregation of nanoparticles increases, a more scattered 2-D pattern is observed. [(**A**) 3 wt% quenched, (**B**) 3 wt%, (**C**) 10 wt%)]

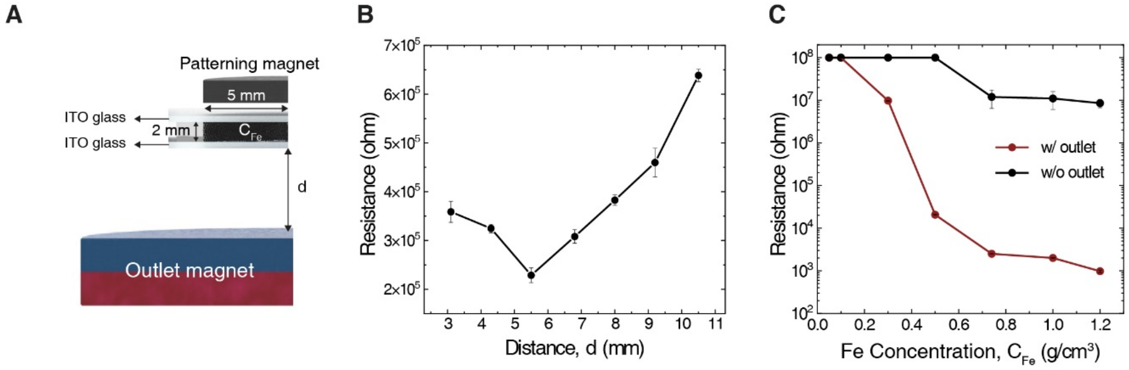


**Fig. S26. The electrical resistance of RAMP with different conditions of magnetic alignment.** (**A**) Schematic of the measurement setup (Patterning magnet: 85 mT, Outlet magnet: 150 mT). (**B**) Changes in electrical resistance of the magnetically aligned particle assembly with increasing distance between the device and the outlet magnet. The conductvie percolation, formed by the nanoparticle assembly, is optimized within the desired patterning area through the balance of magnetic fields between the patterning and outlet magnets. (**C**) Electrical resistance of the RAMP with varying Fe particle concentrations, both with and without the outlet magnet. The electrical pathway forms effectively only in the presence of the outlet magnet.

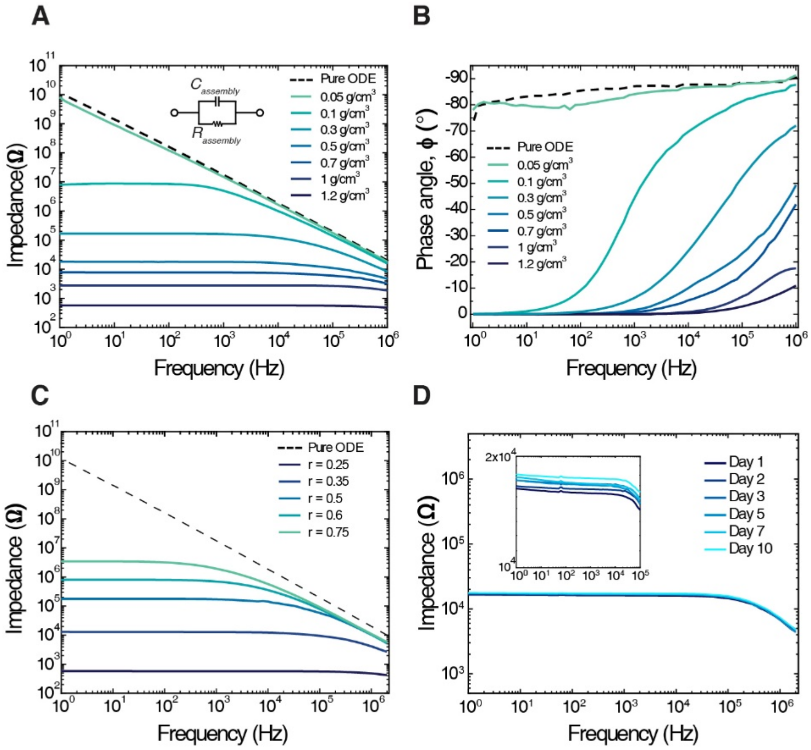


**Fig. S27. Electrical impedance charaterization of RAMP.** (**A** and **B**) Bode plot measured from patterned nanoparticle assembly and the organogel matrix with different particle density per unit volume at room temperature and same patterning area (r = 5 mm, t = 2 mm, concentration of LMOG 7 wt%). (**C**) Bode plot for patterned nanoparticles with varying radius of patterning magnet, maintaining a fixed amount of nanoparticles ($m_{particle}$ = 0.0471 g, t = 2 mm, concentration of LMOG = 7 wt%). (**D**) Impedance changes of RAMP stored at room temperature for 10 days (r = 5 mm, t = 2 mm, $C_{particle}$ = 0.5 g/cm$^3$, $f_{LMOG}$ = 7 wt%).

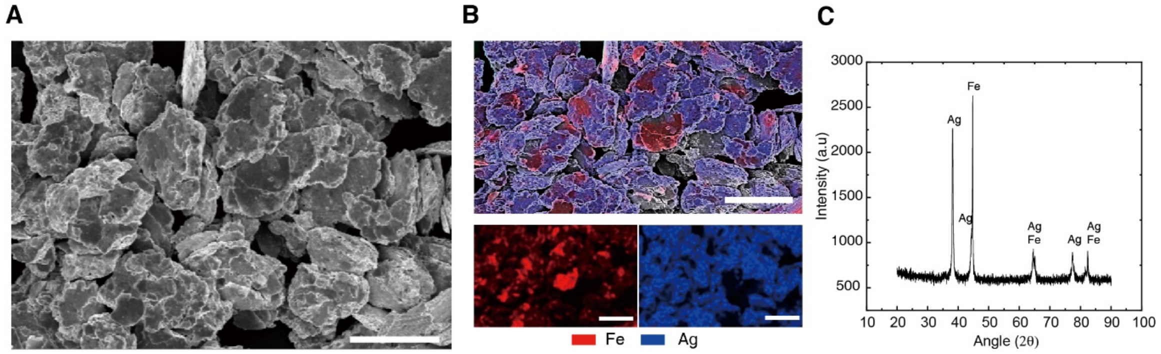


**Fig. S28. Characterization of Ag-coated Fe particles. (scale bar : 30 $\mu$m)** (**A**) SEM and (**B**) EDS images of Ag-coated Fe particles. (**C**) XRD analysis of Ag-coated Fe particles.

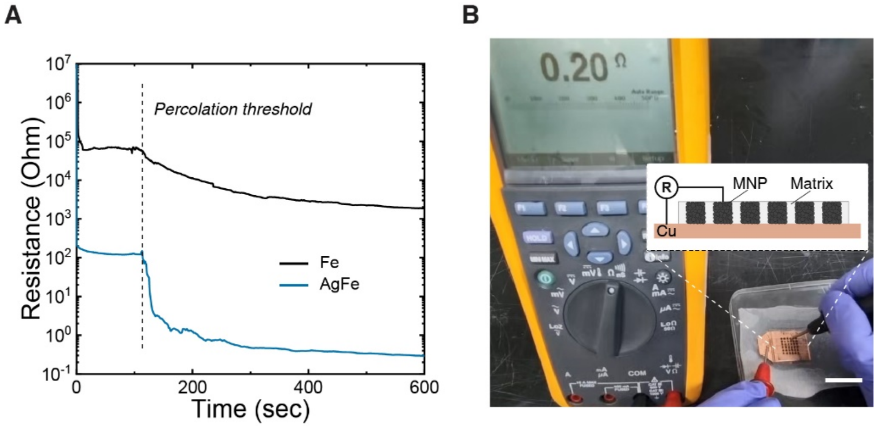


**Fig. S29. Comparison of two systems using different partcles with varying intrinsic resistance.** (**A**) Resistance change in the percolated assembly using different particles (Fe, AgFe) during cooling from 90°C to room temperature. As phase transition is initiated in both samples, a rapid decrease in resistance occurs due to the stabilization of junctions between particles. Their final resistances are determined by the differences in their intrinsic resistances. (**B**) Experimental setup for measuring the resistance of a 1 mm patterned electrode with AgFe particles ($C_{particle}$ = 0.3 g/cm$^3$, $\phi_{LMOG}$ = 10 wt%, sample thickness = 0.5 mm, scale bar: 2 cm).

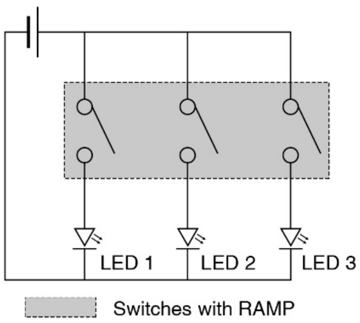


**Fig. S30. An equivalent circuit of reconfigurable non-contact switching demonstration using RAMP system.**

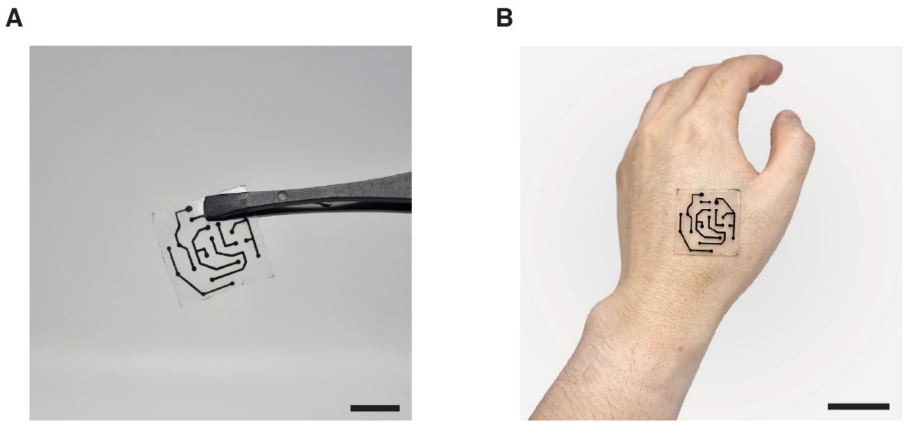


**Fig. S31. Images of circuit-patterned organogel using RAMP.** (**A**) The organogel is free-standing and (**B**) tissue-attachable with high conformability (scale bar: 1 cm).

| Category | Ref | Materials | Mechanism | Reconfigurability | Patternability (resolution) | Stable storage |
|---|---|---|---|---|---|---|
| **Particle assembly for optics/robotics/ printings/ etc** | (*44*) | $Gd_5Si_4$ particles in terpineol | Magnetophoretic chaining during inkjet printing | No | No | Yes |
| | (*45*) | NdFeB particles | Rotating & oscillating magnetic field | No | No | Yes |
| | (*46*) | $Fe_3O_4$ NPs, carbonyl iron particles | Micropatterned magnetophoretic trapping | No (irreversible) | Yes, (~100um, pattern width) | Yes (curing) |
| | (*47*) | Nonmagnetic colloids in ferrofluid | Template-induced magnetic gradient trapping | Yes | Yes (~1-2 um) | No |
| **Particle assembly for conductors** | (*2*) | EGaIn microdroplets in PDMS | Dielectrophoresis-assisted alignment and sintering | No | No | Yes |
| | (*4*) | Ga-coated iron particles | Magnetic alignment + sintering (oxide removal) | No | No | Yes |
| | (*11*) | $Fe_3O_4$-MWNT in oil | Field-induced percolation + hysteresis | Yes | No | Partial |
| | (*13*) | Ni supraparticles | Magnetic jamming & structural memory | Yes | No | Partial |
| | (*15*) | Ag flakes + EGaIn in PVA–Borax | Dry annealing + LM bridging | Yes (self healing) | No | Yes |
| | (*22*) | NiFe particles in PDMS–PBS | AMF-induced oscillation | Yes (self healing) | No | Yes |
| | (*26*) | Ag flakes in hydrogel | Dehydration /rehydration | Yes | No | Yes |
| | (*48*) | Ag–Fe particles in epoxy | Dip-transfer + magnetic chain alignment | No | Yes (~13 um) | Yes |
| | **This work (RAMP)** | **Fe (or Ag-Fe) particles in organogel** | **Magnetic alignment & Phase transition induced particle tightening** | **Yes** | **Yes (~ 500um)** | **Yes** |

**Table S1. Recent field-driven particle assembly system including RAMP approach.**

**Legends for movies**

**Movie S1**

Assembly of magnetic nanoparticles w/ and w/o outlet magnet.

**Movie S2**

Structure of particle percolation

**Movie S3**

FEM simulation of magnetic particle behavior under magnetic field.

**Movie S4**

Reconfigurable electrical switching demonstration using RAMP.

**Movie S5**

The patterning process of RAMP.

**Movie S6**

ACEL operation with self-standing nanoparticle assembly electrode.

**Movie S7**

Demonstration of reconfigurable ACEL display with RAMP.

## REFERENCES AND NOTES


1. J. Zhu, M. Dexheimer, H. Cheng, Reconfigurable systems for multifunctional electronics. *Npj Flex. Electron.* **1**, 8 (2017).

2. F. Krisnadi, L. L. Nguyen, Ankit, J. Ma, M. R. Kulkarni, N. Mathews, M. D. Dickey, Directed assembly of liquid metal–Elastomer conductors for stretchable and self-healing electronics. *Adv. Mater.* **32**, 2001642 (2020).

3. Y. Lu, D. Yu, H. Dong, J. Lv, L. Wang, H. Zhou, Z. Li, J. Liu, Z. He, Magnetically tightened form-stable phase change materials with modular assembly and geometric conformality features. *Nat. Commun.* **13**, 1397 (2022).

4. S. Kim, S. Kim, K. Hong, M. D. Dickey, S. Park, Liquid-metal-coated magnetic particles toward writable, nonwettable, stretchable circuit boards, and directly assembled liquid metal-elastomer conductors. *ACS Appl. Mater. Interfaces* **14**, 37110–37119 (2022).

5. Y.-G. Park, H. S. An, J.-Y. Kim, J.-U. Park, High-resolution, reconfigurable printing of liquid metals with three-dimensional structures. *Sci. Adv.* **5**, eaaw2844 (2019).

6. C. Shi, Z. Zou, Z. Lei, P. Zhu, W. Zhang, J. Xiao, Heterogeneous integration of rigid, soft, and liquid materials for self-healable, recyclable, and reconfigurable wearable electronics. *Sci. Adv.* **6**, eabd0202 (2020).

7. H. Wang, Y. Yao, Z. He, W. Rao, L. Hu, S. Chen, J. Lin, J. Gao, P. Zhang, X. Sun, A highly stretchable liquid metal polymer as reversible transitional insulator and conductor. *Adv. Mater.* **31**, 1901337 (2019).

8. J.-E. Park, H. S. Kang, J. Baek, T. H. Park, S. Oh, H. Lee, M. Koo, C. Park, Rewritable, printable conducting liquid metal hydrogel. *ACS Nano* **13**, 9122–9130 (2019).

9. L. Hu, H. Wang, X. Wang, X. Liu, J. Guo, J. Liu, Magnetic liquid metals manipulated in the three-dimensional free space. *ACS Appl. Mater. Interfaces* **11**, 8685–8692 (2019).

10. S. Choi, S. I. Han, D. Kim, T. Hyeon, D. H. Kim, High-performance stretchable conductive nanocomposites: Materials, processes, and device applications. *Chem. Soc. Rev.* **48**, 1566–1595 (2019).

11. S. W. Lee, S. Baek, S. W. Park, M. Koo, E. H. Kim, S. Lee, W. Jin, H. Kang, C. Park, G. Kim, H. Shin, W. Shim, S. Yang, J. H. Ahn, C. Park, 3D motion tracking display enabled by magneto-interactive electroluminescence. *Nat. Commun.* **11**, 6072 (2020).

12. A. Tricoli, S. E. Pratsinis, Dispersed nanoelectrode devices. *Nat. Nanotechnol.* **5**, 54–60 (2010).

13. X. Liu, H. Tan, C. Rigoni, T. Hartikainen, N. Asghar, S. van Dijken, J. V. Timonen, B. Peng, O. Ikkala, Magnetic field–driven particle assembly and jamming for bistable memory and response plasticity. *Sci. Adv.* **8**, eadc9394 (2022).

14. J. Law, H. Chen, Y. Wang, J. Yu, Y. Sun, Gravity-resisting colloidal collectives. *Sci. Adv.* **8**, eade3161 (2022).

15. Y. Zhao, Y. Ohm, J. Liao, Y. Luo, H.-Y. Cheng, P. Won, P. Roberts, M. R. Carneiro, M. F. Islam, J. H. Ahn, L. M. Walker, C. Majidi, A self-healing electrically conductive organogel composite. *Nat. Electron.* **6**, 206–215 (2023).

16. M. Li, A. Pal, J. Byun, G. Gardi, M. Sitti, Magnetic putty as a reconfigurable, recyclable, and accessible soft robotic material. *Adv. Mater.* **35**, e2304825 (2023).

17. H. Le Ferrand, S. Bolisetty, A. F. Demirörs, R. Libanori, A. R. Studart, R. Mezzenga, Magnetic assembly of transparent and conducting graphene-based functional composites. *Nat. Commun.* **7**, 12078 (2016).

18. J. Lv, G. Thangavel, Y. Xin, D. Gao, W. C. Poh, S. Chen, P. S. Lee, Printed sustainable elastomeric conductor for soft electronics. *Nat. Commun.* **14**, 7132 (2023).

19. C. Gabbett, A. G. Kelly, E. Coleman, L. Doolan, T. Carey, K. Synnatschke, S. Liu, A. Dawson, D. O'Suilleabhain, J. Munuera, E. Caffrey, J. B. Boland, Z. Sofer, G. Ghosh, S. Kinge, L. D. A. Siebbeles, N. Yadav, J. K. Vij, M. A. Aslam, A. Matkovic, J. N. Coleman, Understanding how

junction resistances impact the conduction mechanism in nano-networks. *Nat. Commun.* **15**, 4517 (2024).

20. A. T. Bellew, H. G. Manning, C. Gomes da Rocha, M. S. Ferreira, J. J. Boland, Resistance of single Ag nanowire junctions and their role in the conductivity of nanowire networks. *ACS Nano* **9**, 11422–11429 (2015).

21. A. G. Kelly, D. O'Suilleabhain, C. Gabbett, J. N. Coleman, The electrical conductivity of solution-processed nanosheet networks. *Nat. Rev. Mater.* **7**, 217–234 (2022).

22. R. Xu, G. S. Canon Bermudez, O. V. Pylypovskyi, O. M. Volkov, E. S. Oliveros Mata, Y. Zabila, R. Illing, P. Makushko, P. Milkin, L. Ionov, J. Fassbender, D. Makarov, Self-healable printed magnetic field sensors using alternating magnetic fields. *Nat. Commun.* **13**, 6587 (2022).

23. R. Tutika, A. B. M. Tahidul Haque, M. D. Bartlett, Self-healing liquid metal composite for reconfigurable and recyclable soft electronics. *Commun. Mater.* **2**, 64 (2021).

24. D. Son, J. Kang, O. Vardoulis, Y. Kim, N. Matsuhisa, J. Y. Oh, J. W. To, J. Mun, T. Katsumata, Y. Liu, An integrated self-healable electronic skin system fabricated via dynamic reconstruction of a nanostructured conducting network. *Nat. Nanotechnol.* **13**, 1057–1065 (2018).

25. Y. Ohm, C. Pan, M. J. Ford, X. Huang, J. Liao, C. Majidi, An electrically conductive silver–polyacrylamide–alginate hydrogel composite for soft electronics. *Nat. Electron.* **4**, 185–192 (2021).

26. Y. Ohm, J. Liao, Y. Luo, M. J. Ford, C. Majidi, Reconfigurable electrical networks within a conductive hydrogel composite. *Adv. Mater.* **35**, e2209408 (2023).

27. D. Jung, C. Lim, H. J. Shim, Y. Kim, C. Park, J. Jung, S. I. Han, S.-H. Sunwoo, K. W. Cho, G. D. Cha, Highly conductive and elastic nanomembrane for skin electronics. *Science* **373**, 1022–1026 (2021).

28. E. C. Garnett, W. Cai, J. J. Cha, F. Mahmood, S. T. Connor, M. Greyson Christoforo, Y. Cui, M. D. McGehee, M. L. Brongersma, Self-limited plasmonic welding of silver nanowire junctions. *Nat. Mater.* **11**, 241–249 (2012).

29. E. Ruel-Gariepy, J.-C. Leroux, In situ-forming hydrogels—Review of temperature-sensitive systems. *Eur. J. Pharm. Biopharm.* **58**, 409–426 (2004).

30. C. Wang, H. Wang, B. Wang, H. Miyata, Y. Wang, M. O. G. Nayeem, J. J. Kim, S. Lee, T. Yokota, H. Onodera, On-skin paintable biogel for long-term high-fidelity electroencephalogram recording. *Sci. Adv.* **8**, eabo1396 (2022).

31. T. Li, H. Qi, C. Zhao, Z. Li, W. Zhou, G. Li, H. Zhuo, W. Zhai, Robust skin-integrated conductive biogel for high-fidelity detection under mechanical stress. *Nat. Commun.* **16**, 88 (2025).

32. J. Carvell, E. Ayieta, A. Gavrin, R. Cheng, V. R. Shah, P. Sokol, Magnetic properties of iron nanoparticle. *J. Appl. Phys.* **107**, 103913 (2010).

33. Á. G. García, M. M. Nagelkerke, R. Tuinier, M. Vis, Polymer-mediated colloidal stability: On the transition between adsorption and depletion. *Adv. Colloid Interface Sci.* **275**, 102077 (2020).

34. C. Patrick Royall, S. R. Williams, T. Ohtsuka, H. Tanaka, Direct observation of a local structural mechanism for dynamic arrest. *Nat. Mater.* **7**, 556–561 (2008).

35. K. A. Whitaker, Z. Varga, L. C. Hsiao, M. J. Solomon, J. W. Swan, E. M. Furst, Colloidal gel elasticity arises from the packing of locally glassy clusters. *Nat. Commun.* **10**, 2237 (2019).

36. A. Wang, J. Li, R. Gao, The structural force arising from magnetic interactions in polydisperse ferrofluids. *Appl. Phys. Lett.* **94**, 212501 (2009).

37. R. E. Rosensweig, M. Zahn, R. Shumovich, Labyrinthine instability in magnetic and dielectric fluids. *J. Magn. Magn. Mater.* **39**, 127–132 (1983).

38. G. Yun, S.-Y. Tang, S. Sun, D. Yuan, Q. Zhao, L. Deng, S. Yan, H. Du, M. D. Dickey, W. Li, Liquid metal-filled magnetorheological elastomer with positive piezoconductivity. *Nat. Commun.* **10,** 1300 (2019).

39. G. Yun, S.-Y. Tang, Q. Zhao, Y. Zhang, H. Lu, D. Yuan, S. Sun, L. Deng, M. D. Dickey, W. Li, Liquid metal composites with anisotropic and unconventional piezoconductivity. *Matter* **3**, 824–841 (2020).

40. R. Taherian, Development of an equation to model electrical conductivity of polymer-based carbon nanocomposites. *ECS J. Solid State Sci. Technol.* **3**, M26 (2014).

41. V. J. Anderson, H. N. W. Lekkerkerker, Insights into phase transition kinetics from colloid science. *Nature* **416**, 811–815 (2002).

42. Q. Li, C. W. Kartikowati, S. Horie, T. Ogi, T. Iwaki, K. Okuyama, Correlation between particle size/domain structure and magnetic properties of highly crystalline $Fe_3O_4$ nanoparticles. *Sci. Rep.* **7**, 9894 (2017).

43. D. Serantes, K. Simeonidis, M. Angelakeris, O. Chubykalo-Fesenko, M. Marciello, M. del Puerto Morales, D. Baldomir, C. Martinez-Boubeta, Multiplying magnetic hyperthermia response by nanoparticle assembling. *J. Phys. Chem. C* **118**, 5927–5934 (2014).

44. K. N. Al-Milaji, R. L. Hadimani, S. Gupta, V. K. Pecharsky, H. Zhao, Inkjet printing of magnetic particles toward anisotropic magnetic properties. *Sci. Rep.* **9**, 16261 (2019).

45. Z. Hu, C. Zhang, H. Sun, H. Dai, D. Tang, H. Hu, H. He, L. Wang, H. Zhao, A microstructure enhancement method for hard magnetic particle chains based on magnetic field oscillation sieve. *Mater. Des.* **237**, 112588 (2024).

46. Z. Yang, J. Wei, K. Giżyński, M.-G. Song, B. A. Grzybowski, Interference-like patterns of static magnetic fields imprinted into polymer/nanoparticle composites. *Nat. Commun.* **8**, 1564 (2017).

47. L. He, M. Wang, Q. Zhang, Y. Lu, Y. Yin, Magnetic assembly and patterning of general nanoscale materials through nonmagnetic templates. *Nano Lett.* **13**, 264–271 (2013).

48. H. Yoon, S. Jeong, B. Lee, Y. Hong, A site-selective integration strategy for microdevices on conformable substrates. *Nat. Electron.* **7**, 383–395 (2024).
